# Integrated Network Toxicology, Molecular Dynamics Simulation and In Vitro Assays Identify EGFR, ESR1 and IGF1 as Core Mediators of Bisphenol S-Exacerbated Polycystic Ovary Syndrome

**DOI:** 10.3390/ijms27208961

**Published:** 2026-10-09

**Authors:** Juan Liu, Guolin Ye, Xin Liu, Zhongyuan Deng, Yeling Ma

**Affiliations:** 1 Beijing Key Laboratory of Diagnostic and Traceability Technologies for Food Poisoning, Beijing Center for Disease Prevention and Control, Beijing 100013, China; 2 School of Public Health, Capital Medical University, Beijing 100069, China; 3 School of Agricultural Sciences, Zhengzhou University, Zhengzhou 450001, China; 4 Biology and Agriculture Research Center, University of Science and Technology Beijing, Beijing 100083, China; 5 Medical College, Shaoxing University, Shaoxing 312000, China

**Keywords:** bisphenol S, polycystic ovary syndrome, network toxicology, molecular docking, molecular dynamics simulation

## Abstract

Bisphenol S (BPS), an environmental endocrine disruptor, has been implicated in polycystic ovary syndrome (PCOS), but its underlying molecular targets remain largely uncharacterized. Here, this study integrated network toxicology, molecular simulations and in vitro cellular assays to identify key mediators of BPS-associated PCOS. A total of 199 overlapping target genes between BPS exposure and PCOS were screened from public databases, predicted hub genes were extracted via protein–protein interaction (PPI) network construction and 30 core hub genes were enriched in oxidative stress, hormone response, inflammation, cell-cycle regulation, and PI3K-Akt, FoxO, AGE-RAGE, and endocrine resistance pathways by Gene Ontology (GO) and Kyoto Encyclopedia of Genes and Genomes (KEGG) pathway enrichment analysis. Using molecular docking simulations found that the top 15 hub proteins exhibited favorable binding affinities for BPS, with binding free energies below −5.0 kcal/mol. Molecular dynamics simulations revealed that BPS binding induced only minor conformational perturbations in *EGFR* and *ESR1*, with negligible effects on backbone stability, core domain flexibility, global compactness and surface properties. In contrast, BPS association effectively dampened global backbone fluctuations and the residue-level mobility of *IGF1*, and the intermolecular hydrogen bond strength followed the order *IGF1* > *EGFR* > *ESR1*. In KGN cells, BPS suppressed cell viability and proliferation in a time- and dose-dependent manner while upregulating the protein levels of *EGFR*, *ESR1* and *IGF1*; notably, *IGF1* was most sensitive to low-dose BPS exposure. These findings suggest that BPS may exacerbate PCOS progression by upregulating *EGFR*, *ESR1* and *IGF1* to inhibit granulosa cell growth, and thereby disrupt ovarian oxidative homeostasis, hormone signaling, insulin metabolism and cell-cycle regulation via the PI3K-Akt and FoxO pathways. This study provides a theoretical framework for assessing the reproductive health risks of BPS, and lays a foundation for further experimental validation and population-based epidemiological research.

## 1. Introduction

Polycystic ovary syndrome (PCOS) represents the most prevalent reproductive endocrine and metabolic disorder affecting women of reproductive age worldwide. Its clinical phenotypes encompass two core domains: reproductive abnormalities and metabolic disturbances, which exert long-term adverse impacts on women’s health across the lifespan [1]. Clinically, PCOS is defined by ovulatory dysfunction, hyperandrogenemia and polycystic ovarian morphology, and is frequently complicated by menstrual irregularities, infertility, obesity, insulin resistance, and disrupted glucose and lipid metabolism [2]. Epidemiological data indicate that the global prevalence of PCOS in reproductive-aged women ranges from 6% to 20%, with substantially higher rates reported in populations with obesity, sedentary lifestyles, or high exposure to environmental contaminants. The pathogenesis of PCOS remains incompletely understood. Accumulating evidence suggests that PCOS is not simply a localized ovarian disorder, but a multifaceted syndrome arising from the interplay of genetic susceptibility, neuroendocrine dysregulation, metabolic dysfunction, chronic low-grade inflammation, oxidative stress, and environmental triggers.

The hypothalamic–pituitary–ovarian (HPO) axis constitutes the core regulatory pathway governing female reproductive endocrine homeostasis. Persistent HPO axis dysfunction is widely recognized as a central trigger for PCOS onset, while insulin resistance-driven endocrinometabolic disorders and chronic low-grade inflammation-induced endocrine activation act as pivotal drivers of disease progression. As exogenous contributors, environmental factors act synergistically with genetic predisposition and endogenous functional impairments to amplify endocrine disturbances and accelerate disease progression [3]. Although numerous studies have explored the pathogenesis of PCOS, systematic mechanistic investigations delineating the role of environmental chemicals in disease onset and progression remain scarce.

Endocrine-disrupting chemicals (EDCs) are a broad class of synthetic contaminants pervasive in everyday environments. These compounds interfere with the synthesis, secretion, transport, and metabolism of endogenous hormone, as well as receptor-mediated signal transduction, thereby disrupting reproductive and metabolic endocrine homeostasis and inducing long-term adverse health effects [4]. Given these properties, the potential link between EDCs and PCOS development has received growing research interest. Available evidence reveals that environmental pollutants including bisphenols, phthalate esters, and per- and polyfluoroalkyl substances contribute to PCOS onset and progression by exacerbating hyperandrogenism, worsening insulin sensitivity, and provoking inflammatory responses and oxidative stress [4]. Notably, human biomonitoring studies consistently show higher detection frequencies and concentrations of multiple EDCs in serum or urine samples from PCOS patients compared with healthy controls, implying a potential causal association between EDC exposure and PCOS pathophysiology.

Bisphenols represent a major class of EDCs extensively used in plastic products, food packaging, thermal paper, and industrial materials, resulting in widespread and continuous human exposure throughout the lifespan. Owing to its well-documented reproductive toxicity and endocrine-disrupting properties, bisphenol A (BPA) has been subject to increasing regulatory restrictions worldwide. As a result, BPS has emerged as a widely used alternative to BPA in numerous industrial and consumer applications [5,6]. Chemically, BPS shares a bisphenol backbone with BPA, differing only in the substitution of the dimethylmethylene bridge with a sulfone group. This structural similarity allows BPS to bind to and modulate nuclear receptors, including estrogen receptors and androgen receptors. However, a growing body of evidence demonstrates that BPS is not a safe alternative. BPS exerts estrogenic and anti-androgenic activities as well as metabolic-disrupting effects, and impair ovarian function via multiple mechanisms, including dysregulation of granulosa cell proliferation and apoptosis, disruption of steroid hormone synthesis, and induction of oxidative stress and mitochondrial dysfunction [7,8]. Beyond ovarian dysfunction, BPS exposure has been linked to abnormal folliculogenesis, compromised oocyte quality, and transgenerational reproductive toxicity [9]. At the population level, BPS is frequently detected in human urine, serum and amniotic fluid, underscoring the non-negligible risks arising from chronic cumulative exposure. Importantly, the toxicological profiles of BPS align closely with key pathological features of PCOS, including folliculogenic impairment, hormonal imbalance, and metabolic dysregulation, supporting the hypothesis that BPS may act as an environmental risk factor for PCOS development.

Given that PCOS pathogenesis is governed by intricate networks comprising multiple regulatory factors, molecular targets, and signaling cascades, conventional approaches that focus on individual genes or pathways (e.g., candidate gene association analysis or validation of individual signaling pathways) are insufficient to systematically delineate the molecular mechanisms underlying environmental chemicals induce PCOS. Network toxicology provides a holistic analytical framework to address this limitation. By integrating compound target prediction, disease target identification, network topological analysis, and pathway enrichment analysis, this approach allows dissection of the potential toxic networks of exogenous chemicals within a “compound-target-pathway-disease” paradigm [10,11,12,13,14,15,16]. This strategy not only identifies critical molecular targets but also uncovers crosstalk among distinct signaling pathways, thereby more accurately capturing the complex pathological network underlying PCOS. The subsequent combination with molecular docking and molecular dynamics simulations allows structural and dynamic validation of the binding modes and stability between chemicals and key target proteins. These analyses, which include binding free energy calculation, root-mean-square deviation analysis, and binding pocket residue contribution evaluation, substantially enhance the reliability of mechanistic predictions [17,18]. Such integrative approaches combining network toxicology and computational simulations have been applied to investigate reproductive toxicity induced by various environmental pollutants such as phthalate esters and perfluorinated compounds. However, studies employing this framework to explore the association between BPS exposure and PCOS remain scarce.

**Figure 1:**
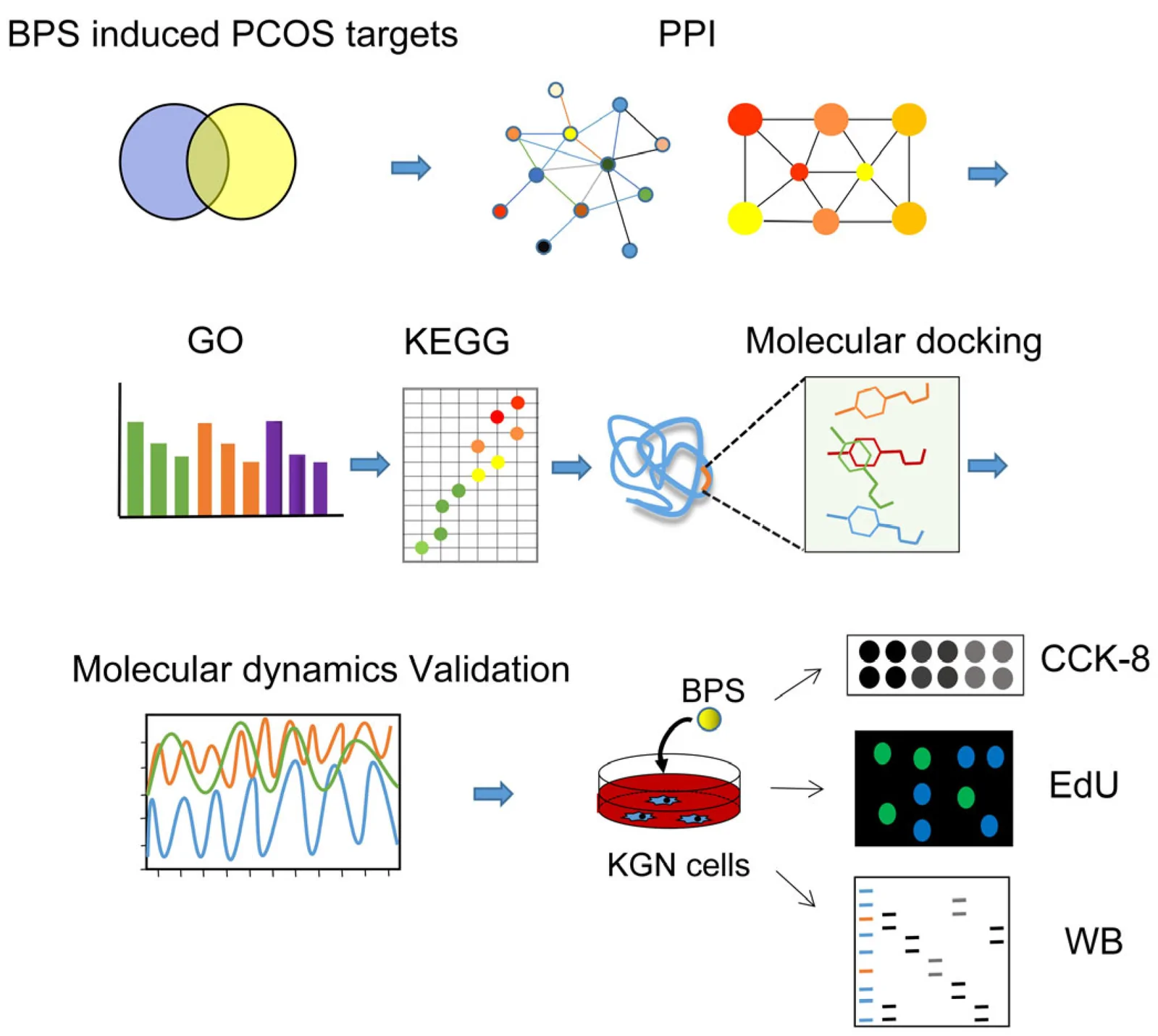
Schematic flowchart illustrating the research framework and experimental workflow of this study.

Against this background, the present study selected BPS as the target environmental chemical to investigate its potential role in PCOS pathogenesis. We first applied network toxicology to screen overlapping targets between BPS exposure and PCOS, and constructed a PPI network to identify core hub genes. We further performed GO enrichment analysis covering biological processes, cellular components and molecular functions, and KEGG pathway enrichment analysis, followed by molecular docking, molecular dynamics simulation, and tissue expression validation using the Human Protein Atlas (HPA) database. Finally, we conducted in vitro molecular experiments to verify the expression of key target proteins (Figure 1). The overall aim of this study is to systematically elucidate the potential molecular mechanisms by which BPS triggers PCOS development, with a focus on core targets, critical signaling pathways, and dynamic binding characteristics. Our findings will provide a theoretical basis for assessing the reproductive health risks of BPS, and lay a foundation for subsequent experimental validation and population-based epidemiological investigations.

## 2. Results

### 2.1. Identification of Overlapping Targets Between BPS and PCOS

**Figure 2:**
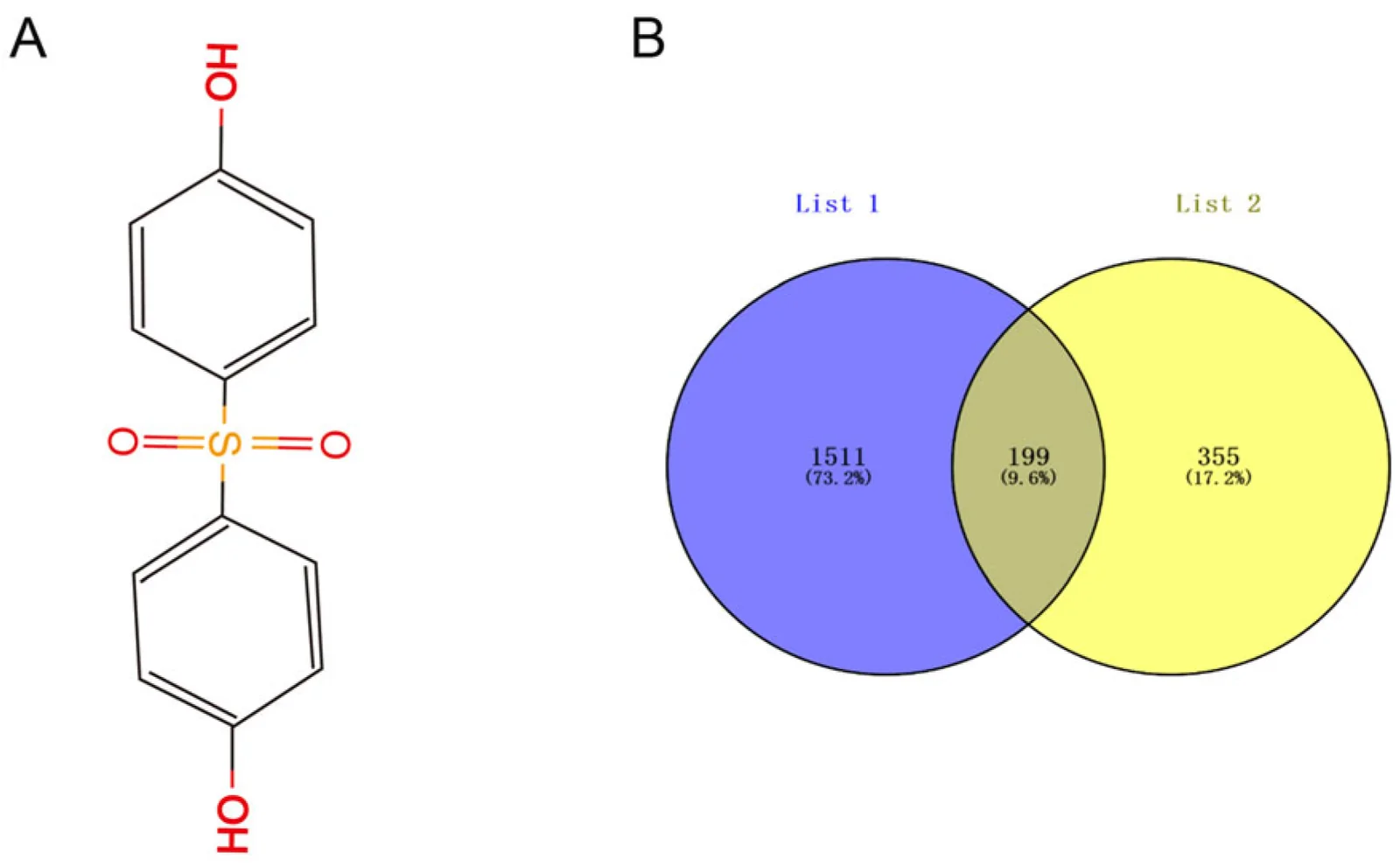
Identification of overlapping targets between BPS exposure and PCOS. (**A**) Molecular structure of BPS. (**B**) Overlapping putative targets shared by BPS and PCOS.

In this study, chemical information for BPS was first retrieved from the PubChem database, including its canonical 2D structure and basic physicochemical properties (Figure 2A and Table 1). Subsequent target intersection analysis identified 1710 PCOS-associated disease targets (List 1) and 554 putative BPS targets (List 2). A total of 199 overlapping core targets were identified, accounting for 35.9% of all BPS targets and 11.6% of all PCOS-related targets (Figure 2B). These overlapping genes were considered critical candidate mediators of BPS-induced reproductive toxicity in the context of PCOS, and were carried forward for subsequent PPI network construction, functional enrichment analysis, and experimental validation.

### 2.2. Construction of the PPI Network and Screening of Hub Target Genes

**Figure 3:**
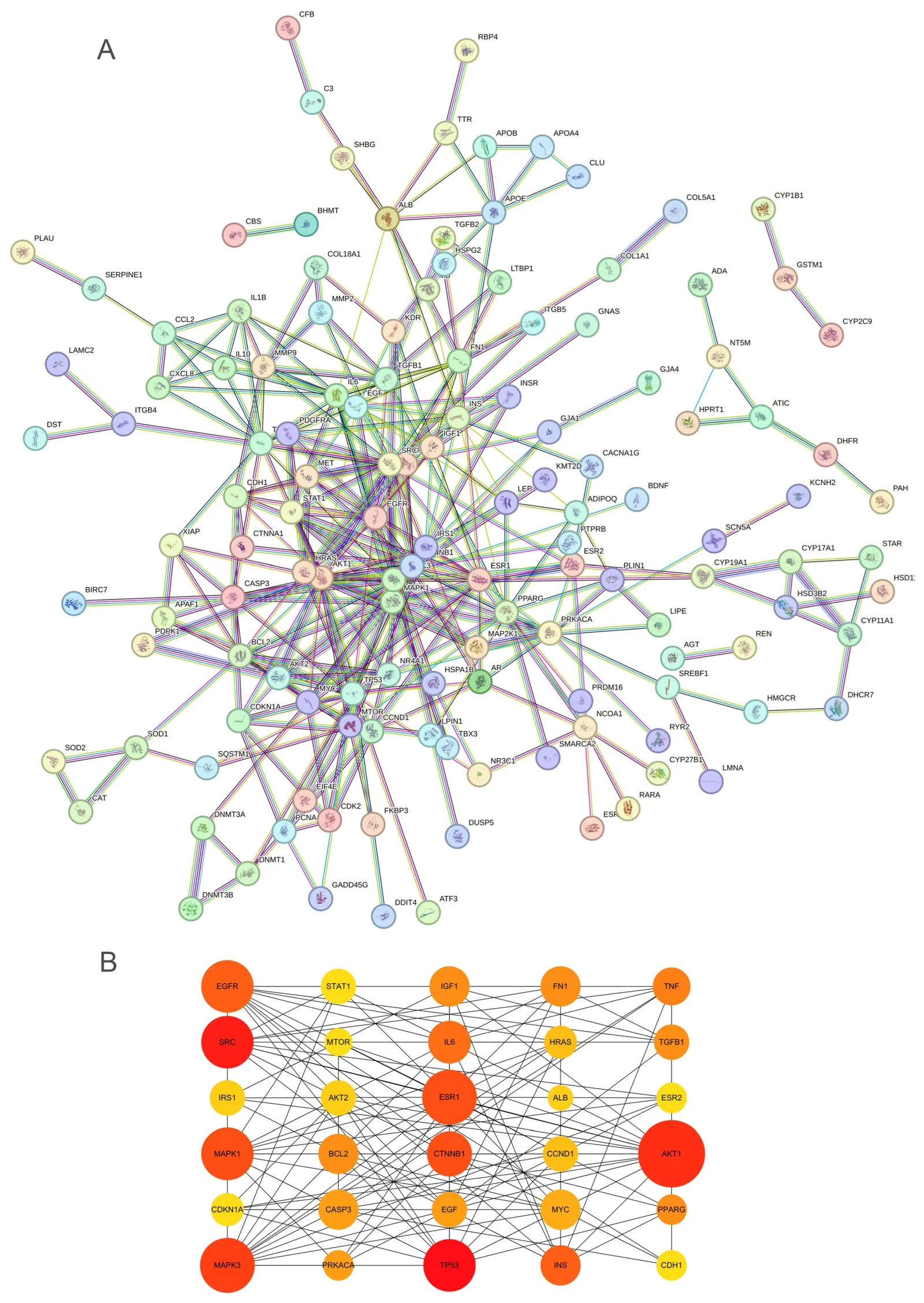
PPI network analysis of overlapping targets between BPS and PCOS. (**A**) Global PPI network constructed in Cytoscape 3.10.4. Each node represents an individual protein, and edges indicate experimentally validated or highly confident predicted protein–protein interactions. Node colors distinguish distinct functional clusters identified by community detection; proteins within the same cluster are functionally associated and tend to be enriched in coherent biological processes. Topological analysis of the full network was performed using the cytoHubba plugin to screen for hub proteins. (**B**) Subnetwork of 30 core hub proteins filtered by cytoHubba. The color gradient from red to yellow corresponds to decreasing topological centrality, and node size is proportional to node connectivity degree.

The 199 overlapping targets shared by BPS and PCOS were submitted to the STRING database to construct a PPI network, which demonstrated dense and extensive functional interactions across the target set. Topological analysis of the resulting network was conducted via the cytoHubba plugin in Cytoscape 3.10.4 software, with node degree selected as the core ranking metric. Based on this metric, the top 30 hub genes were identified. Notably, *TP53, AKT1, ESR1, SRC* and *MAPK3* exhibited the highest node degrees and connectivity, representing the most central nodes in the network (Figure 3). These core genes were therefore defined as key hub targets that potentially mediate the regulatory effects of BPS on PCOS-associated reproductive toxicity.

### 2.3. GO and KEGG Functional Enrichment Analysis of Hub Target Genes

**Figure 4:**
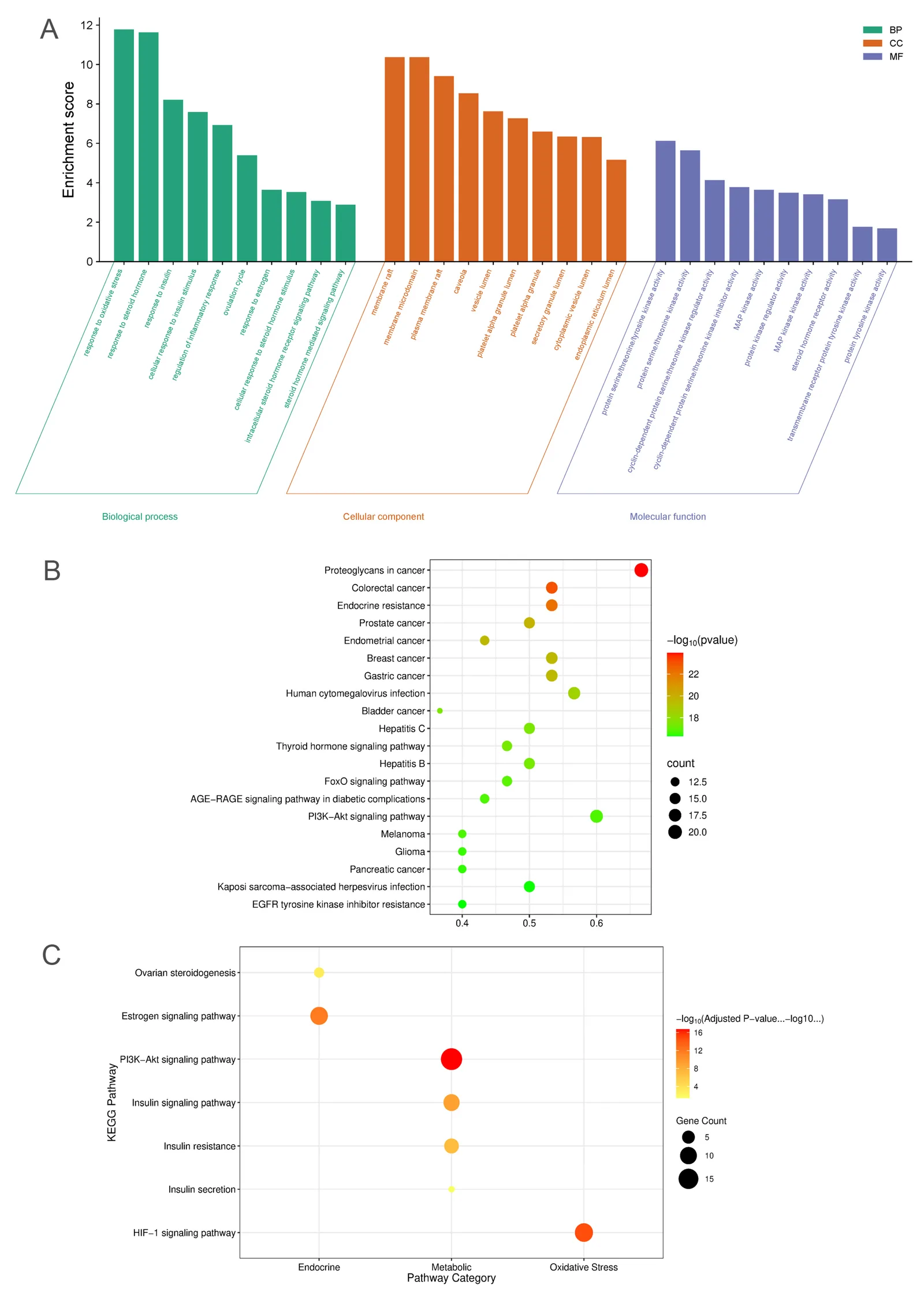
GO and KEGG functional enrichment analysis of the top 30 hub genes shared by BPS and PCOS. (**A**) GO enrichment results across biological process (BP), cellular component (CC), and molecular function (MF) categories. Bar plot showing enrichment scores of three sets of functional terms, distinguished by green, orange and blue bars. (**B**) Bubble plot illustrating the top 20 enriched KEGG pathways derived from the 30 overlapping hub genes. Bubble color indicates −log_10_(*p*-value) (red = higher significance), and bubble size represents the number of enriched genes. (**C**) Bubble plot for pathway-category enrichment, with bubble color denoting -log_10_(*p*-value) and bubble size corresponding to enriched gene count.

GO enrichment analysis was performed on the top 30 predicted core hub targets. This analysis yielded a wealth of information, generating 2308 GO entries, which included 2091 biological processes (BP), 88 cellular components (CC), and 129 molecular functions (MF). In the GO enrichment results, we highlighted the top 10 entries with the highest gene ratio in BP, CC, and MF, respectively, and created a GO enrichment analysis bubble chart. BP analysis revealed significant enrichment in terms associated with oxidative stress response, steroid hormones response, insulin response, inflammatory response regulation, estrogen response, and cell-cycle regulation. For CC analysis, enriched terms covered membrane rafts, membrane microdomains, vesicle lumens, and platelet alpha granules. MF enrichment predominantly involved protein serine/threonine kinase activity, kinase regulatory activity, steroid hormone receptor activity, and transmembrane receptor protein tyrosine kinase activity (Figure 4A). Collectively, these enrichment profiles indicate that BPS may participate in the initiation and progression of PCOS via endocrine disruption, oxidative damage, inflammatory activation and dysregulated signal transduction.

KEGG pathway enrichment generated a comprehensive dataset consisting of 178 entries, which demonstrated that the core hub targets were significantly enriched in multiple signaling pathways, including proteoglycans in cancer, the PI3K-Akt signaling pathway, FoxO signaling pathway, thyroid hormone signaling pathway, and the AGE-RAGE signaling pathway in diabetic complications (Figure 4B). Further filtering against the pathophysiological characteristics of PCOS indicated that endocrine resistance, steroid hormone-related signaling, and insulin regulatory pathways also displayed high enrichment scores (Figure 4C). These findings implied that BPS may contribute to PCOS pathogenesis via the synergistic regulation of multiple signaling cascades.

### 2.4. Molecular Docking Confirmation

Molecular docking simulations were performed to investigate the interactions between BPS and the top 15 screened BPS-PCOS hub targets. Binding energy was adopted as a crucial indicator for evaluating interaction strength, with more negative values reflecting higher binding affinity. A binding energy of less than −5 kcal/mol signifies a strong binding affinity between the ligand and the protein. Using AutoDock Vina 1.2.7 software, molecular docking analysis revealed that all 15 key proteins exhibited high-affinity binding with BPS. The binding energies, ranked from lowest to highest, were as follows: ESR1 (−7.889 kcal/mol, PDB ID:1A52), MAPK3 (−7.255 kcal/mol, PDB ID: 4QTB), EGFR (−7.150 kcal/mol, PDB ID:1M17), AKT1 (−7.019 kcal/mol, PDB ID: 4GV1), SRC (−6.928 kcal/mol, PDB ID: 1FMK), FN1 (−6.592 kcal/mol, PDB ID: 6MFA), TP53 (−6.393 kcal/mol, PDB ID: 1KZY), CASP3 (−6.261 kcal/mol, PDB ID: 4JJE), BCL2 (−6.209 kcal/mol, PDB ID:2W3L), IGF1 (−6.176 kcal/mol, AlphaFold ID: AF_P05019), CTNNB1 (−6.077 kcal/mol, PDB ID: 1JDH), IL6 (−5.993 kcal/mol, PDB ID: 1ALU), MAPK1 (−5.917 kcal/mol, PDB ID: 8AOJ), MYC (−5.571 kcal/mol, AlphaFold ID: AF-P01106), and INS (−5.467 kcal/mol, PDB ID: 5WDM). The majority of the investigated target proteins exhibited Vina binding free energies more negative than −5.0 kcal/mol, satisfying the widely accepted threshold for stable ligand–receptor complex formation. Several targets exhibited binding free energies below −7.0 kcal/mol, reflecting extremely stable ligand–receptor complexes.

**Figure 5:**
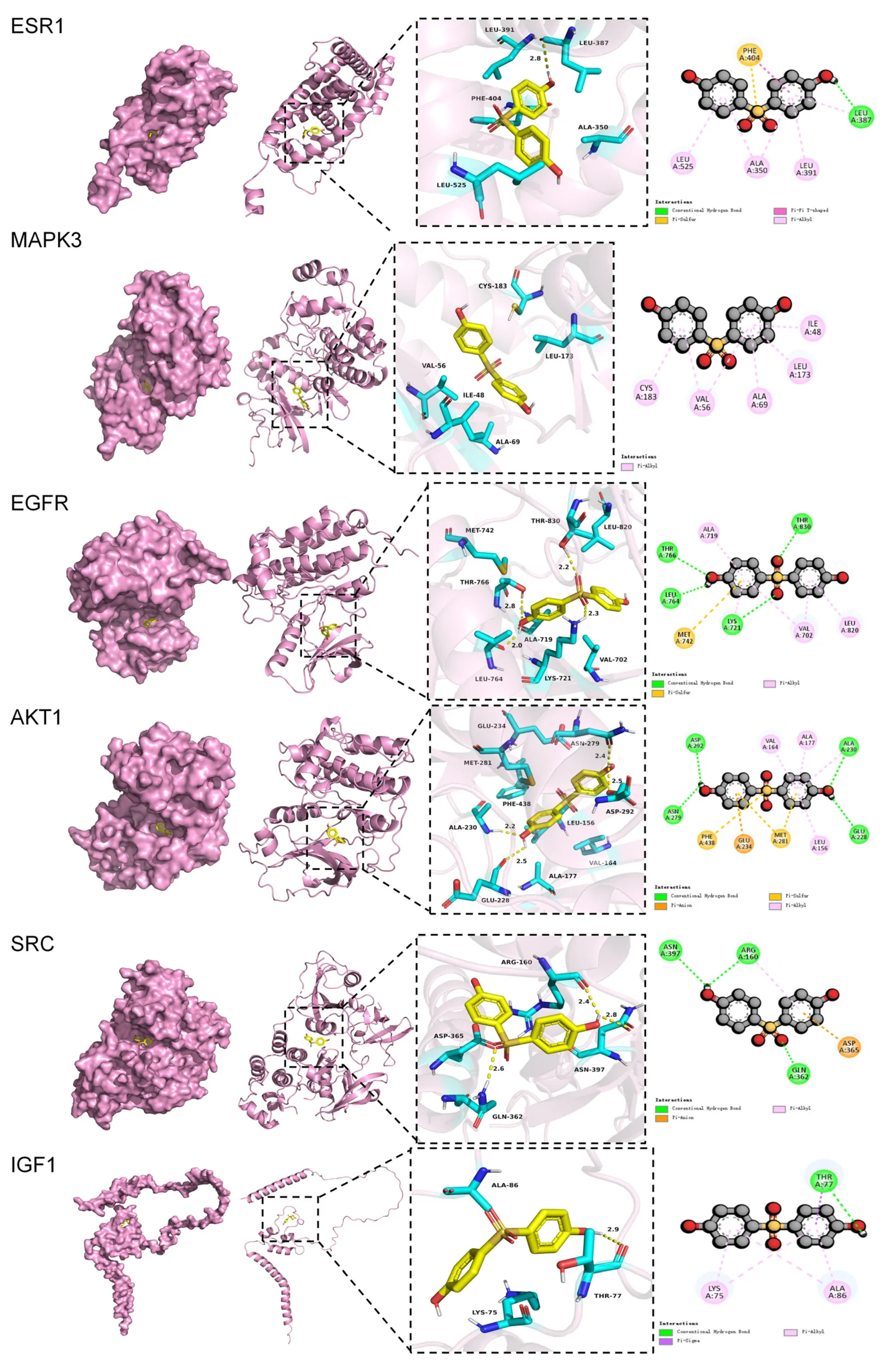
Molecular docking diagrams showing optimal binding conformations of BPS to ESR1, MAPK3, EGFR, AKT1, SRC and IGF-1. Each row shows the docking result of one target protein (ESR1, MAPK3, EGFR, AKT1, SRC and IGF1, from top to bottom). For each protein, panels from left to right present the solvent-accessible surface representation, overall binding mode in cartoon style, magnified view of the binding pocket, and two-dimensional ligand–protein interaction diagram. Receptor proteins are colored magenta. The BPS ligand is shown as yellow stick models, and key amino acid residues lining the binding pocket are highlighted in cyan. In the interaction diagrams, green dashed lines indicate conventional hydrogen bonds, while orange and pink dashed lines represent hydrophobic interactions including π-alkyl, π-sulfur and π-π stacking contacts. All docking simulations were performed using AutoDock Vina 1.2.7.

IGF-1 acts as a key mediator bridging insulin resistance and ovarian dysfunction in PCOS, with its elevated bioavailability exacerbating hyperandrogenemia, arresting follicular maturation, and driving disease progression. Given the pathogenic role of IGF-1 and the lowest binding energy conformations for five protein-BPS complexes, the optimal binding conformations of BPS docked to ESR1, MAPK3, EGFR, AKT1, SRC and IGF-1 were visualized via PyMOL 3.0.3. The overall tertiary structures of receptor proteins and binding pockets of BPS were displayed, and binding pockets were magnified to visualize interaction patterns between BPS and key amino acid residues within pockets (Figure 5). These findings suggest that BPS may exert its effects by binding to these targets, highlighting their considerable significance in the mechanisms underlying BPS-induced PCOS.

### 2.5. Molecular Dynamics (MD) Simulation of Protein–Ligand Complexes

Owing to their prominent topological centrality within the PPI network, together with stable binding conformations and favorable binding affinities toward BPS as verified by molecular docking assays, EGFR, ESR1, and IGF1 were identified as candidate hub targets. Furthermore, all three targets are endogenously expressed in ovarian tissues and play critical regulatory roles in ovarian dysfunction and PCOS pathogenesis. Given that the synergistic modulatory effects of BPS on these key targets in the pathological context of PCOS remain largely uncharacterized, they were ultimately prioritized for follow-up experimental validation.

Molecular dynamics simulations enable visual characterization of conformational dynamics and binding stability of protein–ligand complexes under physiological conditions. Following standard construction workflows in GROMACS 2025.3, 100 ns all-atom MD simulations were implemented for complexes formed by BPS and three predicted core targets (EGFR, ESR1, and IGF1). All simulation systems ran successfully without conformational collapse or complex dissociation throughout the simulation.

**Figure 6:**
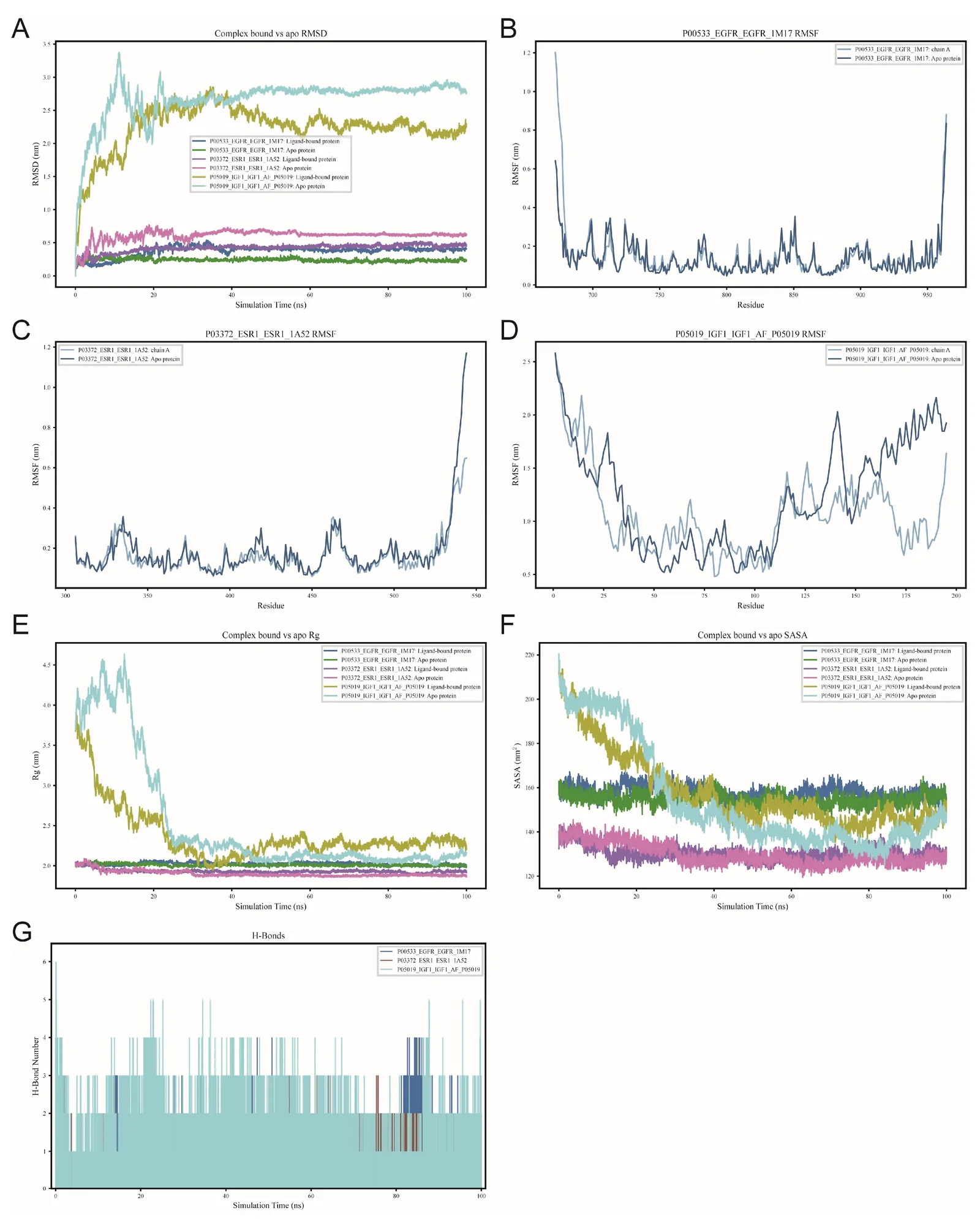
Molecular dynamics analysis of the BPS–protein systems. (**A**) Time evolution of protein Cα RMSD for BPS-bound and apo target proteins during 100 ns simulations. (**B**–**D**) Per-residue RMSF of receptor proteins in the BPS-bound and corresponding apo systems: (**B**) EGFR, (**C**) ESR1, and (**D**) IGF1. (**E**) Protein radius of gyration (Rg). (**F**) Protein solvent-accessible surface area (SASA). (**G**) Number of protein–BPS hydrogen bonds throughout the 100 ns simulation.

Root-mean-square deviation (RMSD) of protein backbones serves as a key metric to assess global conformational stability. Lower RMSD values and reduced fluctuations typically reflect a more stable protein conformation. The six colored curves in Figure 6A represent backbone RMSD profiles of ligand-bound and apo proteins. All apo proteins and BPS-bound complexes reached conformational equilibrium at approximately 20 ns of molecular dynamics simulation. The apo IGF1 system exhibited elevated RMSD values and fluctuated around ~2.8 nm after equilibration, while the BPS-bound IGF1 complex stabilized at ~2.3 nm. In contrast, the EGFR and ESR1 systems showed modest differences in RMSD between apo and BPS-bound forms. The EGFR apo protein maintained low RMSD near 0.2 nm, and its BPS-bound counterpart fluctuated within 0.4–0.5 nm; the ESR1 apo protein stayed at ~0.6–0.7 nm, whereas the BPS-bound ESR1 complex remained around 0.5 nm. These results demonstrate that BPS binding imposes limited perturbation on the overall backbone conformation of EGFR and ESR1, and reduces the global structural fluctuation of IGF1 during simulation.

Residue-level root-mean-square fluctuation (RMSF) serves as an indicator of complex flexibility in simulated dynamic processes, where higher RMSF values correspond to greater flexibility. As shown in Figure 6B–D, RMSF analysis was further conducted to assess the effects of BPS binding on the local conformational flexibility of EGFR, ESR1, and IGF1. For EGFR (Figure 6B), the RMSF trajectories of apo and BPS-bound proteins aligned closely across the structured core region (residues ~700–950), with fluctuation values mostly ranging from 0.05 to 0.35 nm and only sparse low-amplitude peaks. This high degree of overlap indicates that BPS binding causes negligible disruption to the backbone rigidity and local dynamic behavior of the EGFR core domain. Pronounced RMSF elevations were observed at both the N- and C-terminal extremities, in line with the inherent flexibility of protein terminal segments. Specifically, the N-terminal region of BPS-bound EGFR reached a peak RMSF of approximately 1.2 nm, considerably higher than the ~0.65 nm observed for the apo form. A mild increase in RMSF upon ligand binding was also seen at the C-terminus, where both systems retained high intrinsic mobility. Similarly, for ESR1 (Figure 6C), the RMSF profiles of apo and BPS-bound forms were highly comparable across the core domain spanning residues 300–520, with values largely restricted to 0.1–0.35 nm and scattered minor peaks, indicating that BPS ligation exerts minimal impact on the core structural rigidity of ESR1. A sharp RMSF increase occurred at the C-terminal extremity (near residue 550) in both systems; however, the apo protein exhibited a substantially higher peak approaching 1.2 nm, while the BPS-bound complex peaked at only ~0.65 nm, implying that ligand binding partially attenuates the intrinsic flexibility of the ESR1 C-terminal tail. In contrast, the IGF1 system (Figure 6D) showed a more marked divergence in residue flexibility between apo and BPS-bound states. The apo IGF1 protein displayed consistently higher RMSF amplitudes across nearly the entire sequence, with exceptionally high values at the N-terminus (>2.5 nm), a distinct sharp peak around residue 125 (~2.0 nm), and a gradual upward trend along the C-terminal segment. Upon BPS binding, RMSF values decreased across most residues, and these characteristic high-fluctuation peaks were clearly dampened. Collectively, these residue-level flexibility findings are consistent with the global RMSD results, demonstrating that BPS binding induces only limited conformational perturbation to the core structures of EGFR and ESR1, while effectively restraining the local dynamic motions of IGF1.

Radius of gyration (Rg) analysis was performed to track the global compactness and overall size dynamics of EGFR, ESR1, and IGF1 in apo and BPS-bound states over the 100 ns simulation (Figure 6E). For EGFR and ESR1, Rg values remained stable with minimal fluctuation across the entire simulation time for both apo and ligand-bound forms. EGFR systems converged at approximately 2.0 nm, while ESR1 systems stabilized at marginally lower values. The near-perfect overlap between apo and BPS-bound Rg trajectories for both proteins indicates that BPS ligation causes no measurable alteration in global fold compactness. In stark contrast, the IGF1 system exhibited pronounced conformational compaction during the initial simulation phase. Both apo and BPS-bound IGF1 initiated from extended conformations with high Rg values of ~3.8–4.1 nm. The apo IGF1 reached a transient Rg peak of ~4.6 nm around 10 ns, then underwent a continuous collapse until ~30 ns, after which it equilibrated at ~2.1–2.2 nm. BPS-bound IGF1 followed a comparable compaction profile, yet settled at a slightly higher equilibrium Rg (~2.2–2.4 nm) than the apo form, indicating that ligand binding induces a minor expansion of the overall IGF1 structure. These Rg observations align well with the preceding RMSD and RMSF data, collectively confirming that BPS binding exerts little influence on the global structural integrity of EGFR and ESR1, and only modestly modulates the overall conformation of IGF1.

Solvent-accessible surface area (SASA) analysis was performed to evaluate alterations in global solvent exposure and overall surface properties upon BPS binding over the 100 ns molecular dynamics simulation (Figure 6F). For EGFR and ESR1, SASA values remained largely stable throughout the simulation for both apo and ligand-bound states, with only low-amplitude fluctuations around equilibrium levels. BPS-bound EGFR displayed a marginally elevated SASA (~155–165 nm^2^) relative to its apo counterpart (~150–160 nm^2^), and an analogous trend was observed for ESR1, where the ligand-bound form maintained a slightly larger solvent-accessible surface (~130–140 nm^2^) than the apo protein (~125–135 nm^2^). This close overlap between apo and bound trajectories confirms that BPS ligation exerts negligible impact on the global surface topography of these two receptor proteins. In contrast, the IGF1 system exhibited a pronounced decrease in SASA during the initial simulation stage, consistent with the conformational compaction captured by the Rg analysis. The apo IGF1 initiated from an extended conformation with an initial SASA above 220 nm^2^, and declined continuously until approximately 40 ns, after which it stabilized at roughly 135–150 nm^2^. BPS-bound IGF1 followed a comparable compaction profile, yet reached equilibrium at a marginally higher SASA than the apo form, in line with its slightly expanded global dimensions indicated by Rg. Taken together, the SASA results align with the preceding RMSD and Rg data, collectively demonstrating that BPS binding induces minimal perturbation to the overall structural and surface properties of EGFR and ESR1, and only moderately modulates the global conformation and solvent exposure of IGF1.

Hydrogen bonds represent essential intermolecular forces sustaining stable protein–ligand binding; greater hydrogen bond numbers and longer persistence correspond to enhanced binding affinity and conformational stability. As shown in Figure 6G, the IGF1–BPS complex displayed the most abundant and sustained hydrogen bonding interactions across the entire trajectory. The number of intermolecular hydrogen bonds predominantly fluctuated between 2 and 4, with multiple transient peaks reaching 4 to 5, and an initial peak of up to 6 at the onset of simulation. This continuous and frequent hydrogen bond formation indicates strong and extensive polar interactions at the BPS–IGF1 binding interface. For the EGFR–BPS complex, intermolecular hydrogen bonds occurred at a moderate frequency, with bond numbers generally ranging from 2 to 4. A distinct period with sustained 3–4 hydrogen bonds was observed in the middle-to-late simulation stage, whereas the early phase featured more sporadic and intermittent hydrogen bond events. In contrast, the ESR1-BPS complex formed the fewest hydrogen bonds over the simulation. Hydrogen bonding interactions were rarely detected in the first 60 ns, and only emerged more frequently thereafter, with bond counts mostly limited to 1–3. This pattern reflects weaker and less persistent polar interactions between BPS and ESR1 compared with the other two protein targets. These hydrogen bond characteristics align well with the global conformational stability and binding patterns revealed by the preceding RMSD, Rg and SASA analyses, further supporting the differential interaction modes of BPS with EGFR, ESR1 and IGF1.

Collectively, 100 ns all-atom molecular dynamics simulations demonstrate that BPS binding exerts only minor conformational perturbations on EGFR and ESR1, with negligible alterations in backbone stability, core domain flexibility, global compactness and surface properties. In contrast, BPS association effectively dampens global backbone fluctuation and residue-level mobility of IGF1. The intermolecular hydrogen bond strength follows the order IGF1 > EGFR > ESR1.

### 2.6. Validation of EGFR, ESR1, and IGF1 in Ovarian Tissue and KGN Granulosa Cells

Tissue expression profiling of EGFR, ESR1, and IGF1 in ovarian tissue were retrieved from the Human Protein Atlas (HPA) database (Figures S1–S3). Taking EGFR as a representative example, strong protein expression was detected in the follicular cells and ovarian stromal cells of normal ovarian tissues, predominantly localized to the nucleus, with a positivity rate exceeding 75%. Similarly, ESR1 and IGF1 showed constitutive expression in normal ovarian tissues. These expression patterns support critical physiological roles for these genes in the ovary, suggesting that their dysregulation may contribute to PCOS onset and progression.

**Figure 7:**
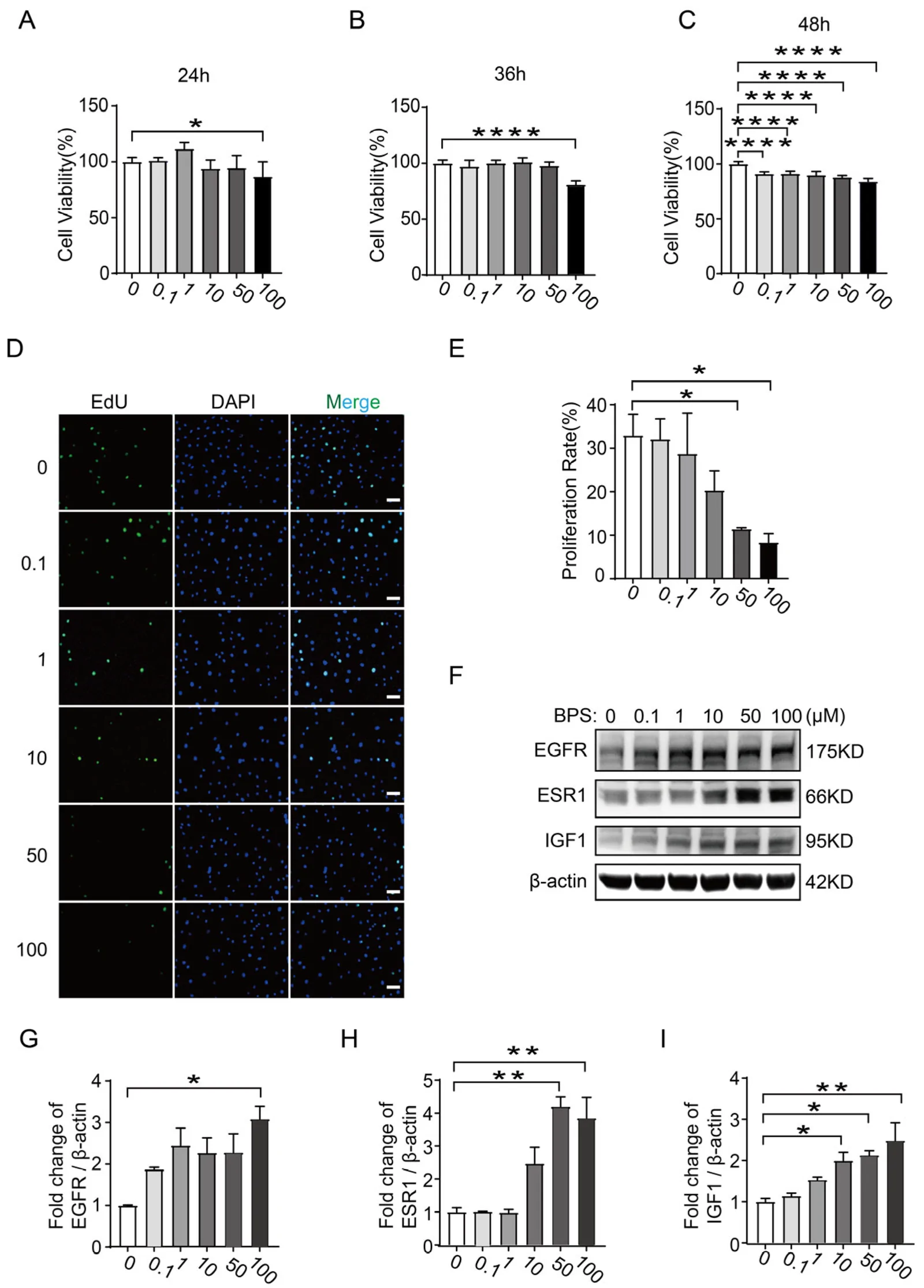
BPS suppresses the viability and proliferation of KGN cells and upregulates the expression of *EGFR*, *ESR1* and *IGF1*. (**A**–**C**) Human ovarian granulosa KGN cells were exposed to gradient concentrations (0, 0.1, 1, 10, 50, and 100 μM, horizontal coordinate) of BPS for 24 h (**A**), 36 h (**B**) and 48 h (**C**), respectively. CCK-8 assay was performed to detect the viability of KGN cells after BPS treatment. (**D**) Representative fluorescence micrographs of EdU staining in KGN cells. Cells were treated with gradient concentrations of the indicated compound (0, 0.1, 1, 10, 50, and 100 μM, in the far-left column) and subjected to EdU proliferation assay. EdU-positive proliferating cells were labeled with Apollo^®^488 (green) (Guangzhou RiboBio Co., Ltd, Guangzhou, China), and cell nuclei were counterstained with DAPI (blue). Merged images are shown in the far-right column. Scale bars indicate 50 μm. (**E**) Quantitative analysis of the cell proliferation rate, calculated as the percentage of Apollo^®^488-positive cells relative to all DAPI-stained nuclei (**F**–**I**) Western blot assay (**F**) and analysis (**G**–**I**) was performed to quantify the protein expression levels of *EGFR*, *ESR1* and *IGF1* in KGN cells after BPS intervention for 36 h. Cells were incubated with BPS at concentrations of 0, 0.1, 1, 10, 50, and 100 μM. β-actin was used as the internal reference protein. The molecular weights of the target proteins are indicated on the right side of each blot. All experiments were independently repeated at least three times. Statistical differences among multiple groups were analyzed by one-way analysis of variance (ANOVA) followed by Tukey’s multiple comparison test. *, *p* < 0.05; **, *p* < 0.01; ****, *p* < 0.0001.

To investigate the regulatory effect of BPS on the pathogenesis of PCOS, human ovarian granulosa cell line KGN was treated with a range of BPS concentrations, and cell viability was detected via the CCK-8 assay. After 24 h of treatment, 100 μmol/L BPS significantly suppressed the viability of KGN cells; this inhibitory effect became more pronounced at 36 h. By 48 h, all tested concentrations of BPS exhibited remarkable inhibitory effects on KGN cell viability (Figure 7A–C). We further performed EdU proliferation assay to verify the impact of BPS on granulosa cell proliferation. The results demonstrated that treatment with 50 μmol/L and 100 μmol/L BPS markedly inhibited the proliferation of KGN cells, which was consistent with the findings of the CCK-8 assay (Figure 7D,E).

To further identify and validate the key target genes mediating BPS-induced PCOS pathogenesis, three predicted candidate genes (*EGFR, ESR1*, and *IGF1*) for protein expression analysis. Following exposure to graded concentrations of BPS, and the protein levels of these three targets in KGN cells were quantified. All three genes showed significant concentration-dependent upregulation at the protein level. Specifically, expression of *EGFR, ESR1*, and *IGF1* were notably higher in the 100 μmol/L BPS group than in the control group. In addition, *IGF1* expression appeared more sensitive to BPS intervention, with significant elevation observed at 10 μmol/L, 50 μmol/L, and 100 μmol/L relative to the control group (Figure 7F–I). Collectively, these data indicate that BPS suppresses the viability and proliferation of ovarian granulosa cells, at least in part via the upregulation of key hub genes including *EGFR, ESR1*, and *IGF1*, which may in turn contribute to PCOS progression.

## 3. Discussion

PCOS is a complex and multifactorial reproductive and metabolic disorder. Accumulating studies have confirmed that chronic exposure to environmental EDSs acts as a critical extrinsic risk factor for PCOS pathogenesis [4]. Nevertheless, the molecular mechanisms by which most prevalent emerging environmental pollutants contribute to PCOS development remain poorly elucidated. In this study, six representative environmental contaminants, namely BPS, p-Phenylenediamine, 1-nitropyrene, 2,6-diiodo-4-nitrophenol, tetrachloro-1,4-benzoquinone, and bis(2-butoxyethyl)-2-hydroxyethyl phosphate, were selected for network toxicology analysis based on solid scientific rationales. These chemicals and their metabolic derivatives are widely distributed in environmental media, and their endocrine and reproductive toxicities have been validated in previous experimental studies. As a primary alternative to traditional bisphenols, BPS is ubiquitously detected in daily consumer products. It possesses estrogenic activity, anti-androgenic effects, and metabolic disruption potential, and impairs ovarian function through multiple mechanisms, including the regulation of granulosa cell proliferation and apoptosis, disturbance of steroid hormone synthesis, and induction of oxidative stress and mitochondrial dysfunction [7,8]. p-Phenylenediamine, an aromatic amine antioxidant derived from rubber materials, produces quinone metabolites that trigger reproductive toxicity via the disruption of endocrine and immune signaling pathways, which has been validated by network toxicology and molecular docking analyses [19]. 1-Nitropyrene is a representative nitro-polycyclic aromatic hydrocarbon formed during incomplete combustion and commonly found in atmospheric particles. Recent in vivo experiments indicate that combined exposure to low-dose of pb^2+^ and 1-nitropyrene impairs the HPG axis through activation of Hippo-dependent pyroptosis [20]. As a well-recognized thyroid-disrupting chemical, 2,6-diiodo-4-nitrophenol interferes with thyroid hormone transport. The resulting disruption of thyroid homeostasis perturbs the neuroendocrine reproductive system and impairs HPG axis homeostasis [21]. Tetrachloro-1,4-benzoquinone, a typical quinone contaminant, induces excessive oxidative stress and ovarian follicular damage [22]. Bis(2-butoxyethyl)-2-hydroxyethyl phosphate is an organophosphate flame retardant. Studies of its structural analogs have shown that long-term exposure to organophosphate esters can dysregulate the transcription of HPG axis-related genes and impair gonadal function [23]. In the present study, we prioritized six candidate chemicals using four core criteria: environmental exposure prevalence, available evidence of reproductive endocrine disruption, toxic potency, and technical feasibility for network toxicology analysis. BPS was ultimately selected as the target chemical for follow-up investigation for three key reasons. First, it exhibits ubiquitous environmental distribution and well-documented human exposure across global populations. Second, abundant direct in vitro and in vivo studies have corroborated its disruptive effects on ovarian granulosa cell function and HPG axis homeostasis. Third, well-annotated protein targets and publicly available protein crystal structures support robust network inference and molecular docking validation. By comparison, Tetrachloro-1,4-benzoquinone and p-Phenylenediamine exhibit moderate reproductive toxicity, whereas direct evidence supporting their endocrine-disrupting potential remains limited. The adverse reproductive outcomes induced by 1-nitropyrene are highly dependent on co-exposure scenarios, introducing variability that complicates single-compound network toxicology analysis. Notably, direct reproductive toxicological data for 2,6-diiodo-4-nitrophenol and bis(2-butoxyethyl)-2-hydroxyethyl phosphate remain scarce. Their potential endocrine risks are largely extrapolated from studies of structural analogs, which substantially limits their suitability for systematic network toxicology validation.

BPS has been frequently detected in a variety of environmental matrices. For surface water, background concentrations in typical lakes and urban river segments range from below the detection limit to tens of ng/L [24,25]. Long-term monitoring data from Taihu Lake report BPS levels of ND–47 ng/L [24], while concentrations in ordinary surface waters across Southeast Asia and southern China fall within 0.15–12 ng/L [25]. BPS accumulates substantially near industrial river stretches and discharge outlets, reaching a maximum of 1.58 μg/L at individual sampling sites [26]. Concentrations in wastewater treatment plant influents vary widely (ND–1249 ng/L), and effluent concentrations decrease markedly after treatment, with a median of 4–10 ng/L [27]. This finding suggests that conventional wastewater treatment can only partially remove BPS rather than eliminate it entirely. Indoor dust represents another major reservoir of BPS, with positive detection reported in global surveys [28]. A domestic investigation of household dust collected from Chinese university students recorded a median BPS concentration of 0.22 μg/g, and global measurements span from below detection to 39.1 μg/g [28]. Incidental ingestion of dust contributes more significantly to BPS exposure in children than in adults. BPS is also present in food matrices, generally at low levels. Fresh food samples obtained from the U.S. market show a median BPS concentration of 0.005 ng/g and a mean value of 0.13 ng/g. BPS can be identified in cereals, canned foods, dairy products and aquatic commodities, though most samples have concentrations below quantification limits [29]. Dietary intake constitutes the primary exposure pathway for the general population, whereas indoor dust ingestion acts only as a secondary route. Biomonitoring studies from the United States and seven Asian countries reveal a high detection rate of urinary BPS, with geometric mean urinary concentrations of 0.030–1.18 μg/L. These urinary data correspond to a median daily intake of 0.004 μg/kg bw/day for adults, and up to 0.02 μg/kg bw/day at the 95th percentile for highly exposed populations. Children display a higher median exposure of approximately 0.009 μg/kg bw/day [30]. Given the ubiquitous exposure to BPS across human populations and higher vulnerability in children, investigating its adverse effects on the reproductive system remains an urgent research priority.

In this study, we integrated network toxicology analysis with molecular simulation approaches to elucidate the mechanisms underlying the involvement of BPS in PCOS pathogenesis. Bioinformatics screening identified 199 overlapping genes between the BPS target gene set and the PCOS-associated gene set. This finding indicates that BPS-elicited ovarian toxicity is driven by the coordinated regulation of multiple functional genes, rather than relying on the modulation of a single molecular target. PPI network analysis further screened 15 pivotal predicted hub genes, including *ESR1*, *MAPK3*, *EGFR*, *AKT1*, *SRC*, *FN1*, *TP53*, *CASP3*, *BCL2*, *IGF1*, *CTNNB1*, *IL6*, *MAPK1*, *MYC*, and *INS*. As predicted central genes in the network, these genes likely play critical roles in mediating the adverse reproductive effects of BPS in ovarian tissue.

Clinically and pathologically, PCOS manifests as a spectrum of typical abnormalities, including hyperandrogenemia, ovulatory dysfunction, insulin resistance, chronic low-grade inflammatory state, and excessive oxidative stress activation [1,2,3]. Consistent with these pathological features, GO enrichment analysis of the intersecting predicted target genes in our study revealed significant enrichment in biological processes involved in oxidative stress response, steroid hormone metabolism, insulin signaling regulation, and inflammatory reaction modulation. These enriched biological pathways highly match the core pathophysiological characteristics of PCOS. Previous clinical and experimental studies have verified that dysregulated oxidative stress status and impaired mitochondrial function in ovarian granulosa cells are key pathogenic drivers of PCOS, adversely affecting follicular maturation, oocyte developmental competence, and homeostasis of the ovarian endocrine microenvironment [31,32]. In support of these findings, existing toxicological studies have confirmed that BPS and its structural analogs trigger excessive reactive oxygen species accumulation, mitochondrial structural damage, and disturbed energy metabolism in granulosa cells. These cellular toxic effects provide a solid biological rationale for the exacerbation of ovarian oxidative damage and metabolic dysfunction following BPS exposure [32,33].

KEGG pathway enrichment analysis was performed to identify key signaling pathways implicated in BPS-mediated PCOS development. The results demonstrated prominent enrichment of the PI3K-Akt, FoxO, AGE-RAGE, and endocrine resistance pathways among the predicted candidate target genes. These results indicate that BPS-associated PCOS progression is driven by the combined effects of dysregulated hormonal signaling, perturbed metabolic homeostasis, and oxidative stress-induced tissue damage, rather than by activation of a single signaling cascade. Functional annotation of these core pathways revealed their distinct biological functions: the PI3K-Akt signaling axis acts as a central regulator of cellular proliferation, glucose metabolism, and insulin signal transduction; the FoxO pathway is a key mediator of apoptosis and endogenous oxidative stress defense; and the AGE-RAGE axis primarily mediates inflammatory responses and metabolic tissue damage [31,34]. Collectively, our results indicate that the pathogenesis of BPS-induced PCOS cannot be attributed solely to estrogen-mimetic effects. Instead, the toxic process is dominated by the synergistic disruption of endocrine function, metabolic homeostasis, and oxidative–inflammatory balance.

Molecular docking analysis was conducted to verify the direct binding interactions between BPS and 15 predicted hub proteins. The results revealed strong binding affinities between BPS and nearly all core target proteins, supporting the structural feasibility of direct molecular interactions between BPS and these functional proteins. As a classic nuclear estrogen receptor, *ESR1* was identified as a core node in the PPI network and showed high binding activity with BPS, which strongly supports the hypothesis that BPS disrupts ovarian endocrine homeostasis through hormone receptor-dependent signaling pathways [35]. In addition, *EGFR*, a typical receptor tyrosine kinase, regulates multiple biological processes including cellular proliferation, differentiation, and growth factor signal transduction [36]. *IGF1* is a key regulatory factor involved in systemic metabolism and insulin-like signaling pathways [37]. Furthermore, 100 ns all-atom molecular dynamics simulations of *ESR1*, *EGFR* and *IGF1* were performed to evaluate the dynamic stability of the BPS-protein complexes. Simulation results confirmed that the composite structures of BPS bound to *EGFR*, *ESR1*, and *IGF1* remained stable throughout the whole simulation process, indicating that these three proteins are the preferential and stable functional targets for BPS to exert ovarian toxic effects. The stable binding characteristics of BPS with *EGFR* and *IGF1* observed in this study further indicates that BPS may interfere with growth factor signaling and metabolic regulatory networks, thereby participating in the pathological progression of PCOS.

Using quantitative in vitro-to-in vivo extrapolation (IVIVE) with human toxicokinetic parameters, the external exposures of BPS translate to steady-state unbound plasma BPS concentrations on the order of low picomolar (pM) range (~5.6–28 pM), representing the biologically available concentration at the target tissue under real human exposure scenarios [38]. Such pM-level concentrations are extremely low and typically insufficient to produce measurable phenotypic or molecular changes in short-term in vitro KGN cell culture assays. The BPS concentration gradient used in our KGN cell experiments spans low nanomolar to micromolar levels. This range matches the 0.1 μM–100 μM doses previously applied in in vitro studies of human granulosa-like KGN cells exploring BPS-induced oxidative stress, biomacromolecular injury, mitochondrial dysfunction, and apoptosis [33,39]. It should be emphasized that the higher concentrations in our study were not intended to directly replicate chronic lifelong human environmental exposure. Instead, they were chosen to activate detectable molecular signaling cascades and characterize the underlying mode of action of BPS in human granulosa cells, a common strategy in mechanistic toxicology research when low environmentally relevant pM concentrations yield no measurable cellular responses within the limited timeframe of in vitro culture.

Consistent with prior reports of BPS-induced endocrine-disrupting toxicological studies [34], the present in vitro experiments demonstrated that BPS exposure inhibited the viability and proliferation of human ovarian granulosa KGN cells in a time and concentration-dependent manner. Significant cytotoxicity was observed at 100 μmol/L BPS following 24 h of treatment, and prolonged exposure further aggravated cell growth suppression, a finding corroborated by EdU incorporation assays. Western blot analysis further revealed that BPS markedly upregulated the protein expression levels of *EGFR*, *ESR1*, and *IGF1* in a dose-dependent fashion. As core pathogenic mediators in PCOS, *EGFR* and *ESR1* are intimately involved in granulosa cell dysfunction and aberrant follicular development [40]. *IGF1*, a key regulator of ovarian metabolism and insulin signaling, exhibited greater sensitivity to low-concentration BPS stimulation, with significantly elevated protein expression detected even at 10 μmol/L [41]. The dysregulated upregulation of these hub proteins reflects the molecular disturbance induced by granulosa cells. Notably, the upregulation of proliferation and metabolism-associated proteins accompanied by suppressed cell proliferation represents a typical compensatory stress response induced by exogenous toxicants. Collectively, these results indicate that BPS mediates granulosa cell growth inhibition and functional disorder by elevating *EGFR*, *ESR1* and *IGF1* protein levels, thereby contributing to BPS-associated granulosa cell pathological changes relevant to PCOS. Nevertheless, this study is limited to in vitro cell models using relatively high BPS concentrations. Further validation with genetic perturbation approaches and in vivo studies is warranted to delineate the precise regulatory mechanisms underlying BPS-driven PCOS-like ovarian injury.

## 4. Materials and Methods

### 4.1. Data Acquisition and Candidate Target Screening

To investigate the toxicological association between environmental pollutant exposure and the development of PCOS, six environmental compounds with reported potential reproductive toxicity were initially selected as candidate agents. These compounds were bisphenol S (BPS, PubChem CID: 6626, CAS: 80-09-1), p-Phenylenediamine (PubChem CID: 7814, CAS: 106-50-3), nitro-polycyclic aromatic hydrocarbons (represented by 1-Nitropyrene, PubChem CID: 21694, CAS: 5522-43-0), 2,6-Diiodo-4-nitrophenol (PubChem CID: 9370, CAS: 305-85-1), tetrachloro-1,4-benzoquinone (PubChem CID: 8371, CAS: 118-75-2), and bis(2-butoxyethyl)-2-hydroxyethyl phosphate (PubChem CID: 132967161, CAS: 1477494-86-2).

The ProTox 3.0 and ChEMBL databases were employed to systematically evaluate the toxicological and bioactivity profiles of the six candidates. ProTox 3.0 was used to predict acute toxicity, organ-specific toxicity, and adverse toxic endpoints, while ChEMBL was queried to retrieve annotated bioactivity data against human molecular targets. After comprehensive assessment of overall toxic potency and the strength of evidence linking each compound to reproductive endocrine disruption, BPS was determined as the priority environmental toxicant for subsequent mechanistic investigation.

Potential molecular targets of BPS were predicted using two complementary in silico tools: Swiss Target Prediction and the Similarity Ensemble Approach (SEA) databases [42,43]. Swiss Target Prediction identifies putative targets based on two-dimensional and three-dimensional structural similarity to known bioactive ligands, and SEA integrates multiple similarity metrics to improve prediction robustness. The UniProt database was adopted to standardize gene nomenclature for all predicted target proteins, with the organism limited to *Homo sapiens* to ensure species consistency [44]. Following removal of invalid entries, uncharacterized proteins, and duplicate gene symbols, a total of 554 valid potential targets of BPS were retained.

Disease targets associated with PCOS were mined from three authoritative databases: GeneCards, Online Mendelian Inheritance in Man (OMIM), and NCBI Gene Expression Omnibus (GEO) [45]. The search was conducted using the keywords “Polycystic Ovary Syndrome” and “PCOS”, and only entries supported by experimental or clinical evidence of functional or causal association with PCOS were included. After gene symbol standardization and deduplication, a dataset comprising 1710 PCOS-related disease targets was constructed.

The Venny 2.1.0 online platform was applied to generate Venn diagrams and identify overlapping targets between the BPS target set and the PCOS disease target set. Ultimately, 199 intersecting targets were identified, which were defined as core candidate targets that potentially mediate the toxic effects of BPS in the progression of PCOS.

### 4.2. Protein–Protein Interaction Network Construction and Hub Gene Identification

Following the identification of 199 overlapping targets genes, a protein–protein interaction (PPI) network was constructed using the STRING database (https://string-db.org/) [46]. The organism was set to *Homo sapiens*, and the minimum interaction confidence threshold was defined as the highest confidence level (≥0.900) to ensure high reliability of the retrieved protein interactions. Isolated nodes without interactive partners were excluded from the network to focus on the functionally interconnected gene cluster. The interaction data exported from STRING were subsequently imported into Cytoscape 3.10.4 software for network visualization and topological analysis [17]. The cytoHubba plugin was adopted to assess the topological importance of each node in the network. Genes were ranked by their Degree score, which represents the number of direct interconnections for a given node, and the top 30 genes with the highest Degree values were screened as hub target genes.

### 4.3. Gene Ontology and KEGG Pathway Enrichment Analyses

Based on the 30 predicted hub genes identified, Gene Ontology (GO) functional enrichment analysis and Kyoto Encyclopedia of Genes and Genomes (KEGG) pathway enrichment analysis were performed using the Bioinformatics online platform (https://www.bioinformatics.com.cn/). Official gene symbols of the hub genes were used as input, with *Homo sapiens* specified as the reference species and the complete set of human protein-coding genes adopted as the background gene set for enrichment calculation. Enrichment significance was evaluated via hypergeometric test, and a *p*-value < 0.05 was set as the cutoff for statistical significance.

GO enrichment results were categorized into three core ontological domains: biological process (BP), cellular component (CC), and molecular function (MF), which annotate the gene set from the dimensions of biological function, subcellular localization, and molecular activity, respectively. For KEGG pathway analysis, all significantly enriched pathways were sorted in ascending order of *p*-value. The top 20 pathways with the strongest statistical significance were retained for graphical presentation to provide an overall landscape of the involved signaling and metabolic pathways. Given the study’s focus on endocrine and metabolic regulation, pathways related to oxidative stress, estrogen regulation, and endocrinometabolic homeostasis were further manually curated from the full KEGG results and highlighted in the result section for in-depth biological interpretation.

### 4.4. Molecular Docking Analysis of Predicted Core Target Proteins

Molecular docking simulations were performed to predict the binding modes between the small-molecule ligand and target proteins (e.g., enzymes and transcription factors). To simulate the formation of stable ligand–protein complexes and support subsequent toxicological mechanism elucidation, a semi-flexible docking strategy was adopted, and all docking calculations were carried out using AutoDock Vina 1.2.7 [47,48]. Bisphenol S (BPS; PubChem CID: 6626) was used as the ligand, and 15 core predicted target proteins (ESR1, MAPK3, EGFR, AKT1, SRC, FN1, TP53, CASP3, BCL2, IGF1, CTNNB1, IL6, MAPK1, MYC, and INS) served as receptors.

Three-dimensional structures of the predicted target proteins were retrieved from the RCSB PDB database (experimentally resolved crystal structures) or the AlphaFold Protein Structure Database (computationally predicted structural models). Initial structural preprocessing was carried out in PyMOL 3.0.3. For crystal structures, crystallographic water molecules, non-target co-crystallized ligands, and other redundant components were removed, while for predicted structures, intact polypeptide chains were retained directly. The preprocessed protein structures were subsequently imported into AutoDock Tools 1.5.7, where full hydrogen atoms were added, Gasteiger atomic charges were computed and assigned, non-polar hydrogens were merged into their covalently linked heavy atoms, and atom types and bond orders were corrected. Finally, the prepared receptor structures were exported in PDBQT format as docking inputs.

The initial three-dimensional structures of BPS were obtained from the PubChem database or converted from two-dimensional structures. Molecular mechanics energy minimization was performed using Chem3D 16.0 with the MMFF94 (Merck Molecular Force Field 94) force field, a widely accepted standard for small-molecule conformation optimization [49]. The conjugate gradient method was used as the optimization algorithm, with a convergence threshold of 0.001 kcal/(mol·Å) and a maximum of 1000 iterations until the system energy gradient satisfied the convergence criterion. The optimized ligands were then imported into AutoDock Tools 1.5.7, assigned Gasteiger atomic charges, configured with all rotatable single bonds as flexible torsions, and exported in PDBQT format.

Docking grids were defined following a hierarchical strategy tailored to the structural source of each receptor protein. For target proteins retrieved from the RCSB PDB database, the docking grid was centered on the protein’s canonical active site or ligand-binding pocket, which was identified according to the original ligand-binding region annotated in the crystal structure. For target proteins obtained from the AlphaFold Protein Structure Database, where no co-crystallized native ligands were available as spatial reference, the canonical ligand-binding pocket was predicted using the CurPocket algorithm based on protein surface curvature and accessible surface area [50]. This algorithm outputs 5 candidate pockets ranked by binding potential score by default, and the geometric centroid of the top-ranked candidate cavity was adopted as the grid center. For all receptors, the grid dimensions were set uniformly to 20 Å × 20 Å × 20 Å with a default grid spacing of 0.375 Å, the standard precision for the AutoDock suite. This size was moderately expanded relative to the pocket volume to fully cover the entire binding cavity, ensuring that BPS could freely explore potential binding poses within the functional region.

Docking calculations were executed under the aforementioned semi-flexible strategy. Core parameters of AutoDock Vina 1.2.7 were set as follows: exhaustiveness was set to 32 to ensure comprehensive conformational sampling and high result reproducibility; 9 binding conformations were generated per pocket; the energy range was set to 3 kcal/mol; and the RMSD threshold for conformational clustering was 1.0 Å. Binding free energy was calculated using the software’s built-in empirical scoring function, which evaluates binding stability by integrating van der Waals interactions, hydrogen bonding, electrostatic interactions, and conformational entropy. Docking runs were performed independently for each candidate pocket, and the binding mode associated with the lowest minimum binding free energy across all pockets and conformations was selected as the most probable ligand–target protein interaction pattern.

The optimal docking results were visualized in three dimensions using PyMOL 3.0.3 to display the spatial orientation of the ligand within the binding pocket, the distribution of key amino acid residues of the target protein, and the overall conformation of the complex. Two-dimensional ligand–receptor interaction diagrams were generated using Discovery Studio, annotating the types and distances of non-covalent interactions including hydrogen bonds, hydrophobic interactions, π-π stacking, and salt bridges, to intuitively present the details of intermolecular interactions.

### 4.5. Molecular Dynamics Simulations of Protein-BPS Complexes

All-atom molecular dynamics (MD) simulations were performed using the GROMACS 2024.2 software package, following the standard high-performance parallel simulation protocol established by Van Der Spoel et al. (2005) [51]. The AMBER99SB-ILDN force field was applied to assign topological parameters to all target proteins. For the BPS ligand, the general AMBER force field (GAFF) was adopted, with partial atomic charges calculated via the AM1-BCC method using the Antechamber module. The TIP3P water model was adopted for the solvation of both ligand-free apo proteins and BPS-bound complexes.

Each solute was centered in a rhombic dodecahedral periodic box with a minimum solute-to-box distance of 1.2 nm, and then solvated with TIP3P water molecules. To replicate physiological aqueous conditions, Na^+^ and Cl^−^ counterions were added to neutralize the net electrostatic charge of each system, and additional NaCl was introduced to reach a final physiological concentration of 0.15 M. The detailed composition of each system is as follows: the BPS–EGFR system contained 15,774 water molecules, 48 Na^+^ ions, and 49 Cl^−^ ions, with an initial box volume of 536.087 nm^3^; the BPS–ESR1 system contained 24,705 water molecules, 79 Na^+^ ions, and 72 Cl^−^ ions, with an initial box volume of 793.762 nm^3^; the BPS–IGF1 system contained 100,925 water molecules, 283 Na^+^ ions, and 303 Cl^−^ ions, with an initial box volume of 3127.940 nm^3^. The corresponding apo protein systems had slightly different numbers of water molecules (15,779 for EGFR, 24,708 for ESR1, 100,935 for IGF1) and the same ion counts as their BPS-bound counterparts. The imbalance between Na^+^ and Cl^−^ numbers arises from the counterions required for neutralization of the intrinsic net charge of each protein.

Prior to production simulations, all systems underwent stepwise energy minimization and equilibration to ensure thermodynamic stability. First, energy minimization was performed using the steepest descent algorithm to eliminate unfavorable steric clashes and reduce the potential energy of the system, with a convergence criterion of maximum atomic force below 1000 kJ·mol^−1^·nm^−1^. Subsequently, two successive equilibration stages were carried out with a 2 fs time step, during which position restraints with a force constant of 1000 kJ·mol^−1^·nm^−2^ were applied to the protein backbone to preserve the initial conformation. The first stage consisted of 2 ns NVT ensemble equilibration at 298 K, with temperature controlled by a Berendsen thermostat (coupling time τT = 0.1 ps). The second stage was 2 ns NPT ensemble equilibration at 298 K and 1.0 bar, with pressure regulated by a Berendsen barostat. All bonds involving hydrogen atoms were constrained using the LINCS algorithm throughout all simulation stages.

Production MD simulations were conducted for 100 ns in the NPT ensemble at 298 K and 1.0 bar, with a 2 fs integration time step. The Nosé–Hoover thermostat (coupling time τT = 1.0 ps) and the isotropic Parrinello–Rahman barostat (coupling time τP = 2.0 ps; isothermal compressibility = 4.5 × 10^−5^ bar^−1^) were employed for precise temperature and pressure control. Long-range electrostatic interactions were calculated using the particle-mesh Ewald (PME) method with a real-space cutoff of 1.2 nm. Van der Waals interactions were truncated at 1.2 nm, and long-range dispersion correction was applied to both energy and pressure calculations to compensate for truncation artifacts. Periodic boundary conditions were imposed in all three dimensions throughout all simulation stages.

All structural analyses were performed on the full 0–100 ns production trajectory at a sampling interval of 10 ps, corresponding to 10,001 frames per system. Prior to calculation, periodic boundary artifacts were removed, each complex was re-centered in the periodic box, and the trajectory was aligned via least-squares fitting of the protein Cα atoms. The root-mean-square deviation (RMSD) of protein Cα atoms was calculated to assess the global structural stability of the protein backbone. Per-residue root-mean-square fluctuation (RMSF) was computed from all protein atoms after alignment to characterize the local conformational flexibility of each amino acid residue. The mass-weighted radius of gyration (Rg), derived from all protein atoms, was used to evaluate the overall compactness of the protein. The solvent-accessible surface area (SASA) was determined with a 0.14 nm solvent probe based on all protein atoms, reflecting the solvent exposure level of the protein surface. The occupancy of protein–BPS hydrogen bonds was analyzed using donor and acceptor atoms from both moieties, with standard geometric criteria (donor–acceptor distance ≤ 0.35 nm and hydrogen–donor–acceptor angle ≤ 30°), to validate the stability of intermolecular interactions.

Binding free energies were estimated using the molecular mechanics/Poisson–Boltzmann surface area (MM/PBSA) method, as implemented in gmx_MMPBSA v1.5.0.3 with a single-trajectory protocol. Twenty-six snapshots were extracted from the final 10 ns (90–100 ns) of each production trajectory at 0.4 ns intervals. The Poisson–Boltzmann (PB) calculation was performed with mbondi2 atomic radii, an ionic strength of 0.15 M, an internal dielectric constant of 1.0, an external dielectric constant of 80.0, a solvent probe radius of 1.4 Å, a grid spacing of 0.5 Å, and a fill ratio of 4.0 at 298 K. Per-residue energy decomposition was carried out to quantify the contribution of individual residues to total binding affinity. No configurational entropy term was included in the calculations.

### 4.6. Tissue Expression Profiling of EGFR, ESR1 and IGF1 in Ovarian Tissue from HPA Database

Immunohistochemical expression profiles of five core hub proteins (EGFR, ESR1 and IGF1) in normal ovarian tissues were retrieved from the Human Protein Atlas (HPA) database [52], to verify the basal expression and subcellular localization of these key molecules in ovarian tissue at the proteomic level.

The HPA Tissue Atlas provides systematically validated immunohistochemical data across a broad panel of normal human tissues, with all specimens collected from histologically confirmed healthy donors. For normal ovarian tissue, each target protein is represented by 3 to 6 independent donor samples with preserved histological architecture. All primary antibodies used in the HPA dataset have undergone rigorous specificity validation, including Western blotting, control staining assays, and gene silencing verification, ensuring the reliability and accuracy of protein detection.

Staining results were evaluated using the standardized semi-quantitative scoring system adopted by the HPA consortium. Two core parameters were assessed for each sample: staining intensity (classified as negative, weak, moderate, or strong) and the proportion of positively stained cells (categorized as <25%, 25–75%, or >75%). The overall expression level of each protein was further stratified into four grades (not detected, low, medium, high) based on the integrated scoring of intensity and positive cell fraction. Subcellular localization patterns (cytoplasmic, membranous, and nuclear) of each target protein were also extracted according to official HPA annotations. Consensus results across all donor samples were summarized to confirm the basal presence of these hub proteins in ovarian tissue, providing histological support for subsequent toxicological mechanism analysis.

### 4.7. KGN Cell Culture

The human ovarian granulosa tumor cell line KGN was purchased from Beijing Xiecheng Biotechnology Co., Ltd. (Beijing, China). Cell identity was confirmed by morphological observation and growth curve validation after thawing. All experiments used cells between passage 3 and 25 to ensure stable proliferation and a consistent phenotype. Routine mycoplasma testing was performed every 2 weeks via Hoechst 33258 fluorescence staining, and only mycoplasma-negative cell batches were used for formal assays.

Cells were routinely maintained in DMEM/F12 medium (Thermo Fisher Scientific, Shanghai, China) supplemented with 10% fetal bovine serum (Gibco, Auckland, New Zealand), 100 U/mL penicillin and 100 μg/mL streptomycin (both from Thermo Fisher Scientific, Shanghai, China). Cultures were incubated at 37 °C in a humidified 5% CO_2_ atmosphere. When reaching 70–80% confluence, cells were dissociated with 0.25% trypsin–EDTA (Thermo Fisher Scientific, Shanghai, China) and passaged at a 1:3 to 1:4 split ratio. All functional experiments were performed with cells in the logarithmic growth phase.

### 4.8. Experimental Grouping and BPS Intervention Settings

Bisphenol S (BPS, purity ≥ 99%, Sigma-Aldrich, St. Louis, MO, USA) was dissolved in dimethyl sulfoxide (DMSO) to prepare a 100 mM stock solution, aliquoted and stored at −20 °C in the dark. Working solutions were freshly diluted to target concentrations with complete medium immediately before use. A vehicle control group was set in parallel, receiving an equal volume of DMSO as the highest-dose BPS group, with a final DMSO concentration below 0.1% (*v*/*v*) to rule out solvent toxicity. Five BPS treatment groups were set at final concentrations of 0.1 μM, 1 μM, 10 μM, 50 μM and 100 μM, covering both environmentally relevant exposure levels and pathophysiologically active doses.

### 4.9. Cell Counting Kit-8 Assay

Cell viability was assessed using the Cell Counting Kit-8 (CCK-8) assay (MedChemExpress, Shanghai, China). The reagent was stored at 4 °C protected from light and equilibrated to room temperature for 30 min before use to avoid temperature deviation that interferes with the chromogenic reaction. KGN cells in the logarithmic growth phase were digested with 0.25% trypsin-EDTA, resuspended in complete culture medium and counted with a hemocytometer. The cell suspension was adjusted to a density of 5 × 10^4^ cells/mL and seeded into 96-well plates at 100 μL per well, the suspension was gently swirled at intervals during seeding to prevent cell sedimentation and ensure consistent cell loading per well. Plates were incubated at 37 °C in a humidified 5% CO_2_ atmosphere for 24 h to allow full cell adhesion.

After adhesion, the medium was replaced with serum-free DMEM/F12 for 12 h to synchronize cells at the G0/G1 phase. Cells were then exposed to graded concentrations of BPS (0.1 μM, 1 μM, 10 μM, 50 μM and 100 μM) diluted in serum-free medium, with a vehicle control group set in parallel that received an equal volume of DMSO. Six replicate wells were allocated to each treatment group, and cell viability was detected at 24 h, 36 h and 48 h after BPS administration, with each time point assayed on an independently prepared plate.

At each predetermined time point, 10 μL of CCK-8 reagent was slowly added along the inner wall of each well to avoid generating air bubbles that would distort absorbance readings. Plates were returned to the incubator and incubated at 37 °C for exactly 2 h in the dark, after which the absorbance at 450 nm (OD_450_) of each well was measured with a microplate reader. Relative cell viability was calculated using the formula: cell viability (%) = [(OD_treatment_ − OD_blank_)/(OD_control_ − OD_blank_)] × 100%, where OD_blank_ represents the absorbance of wells containing only culture medium and OD_control_ represents the absorbance of the vehicle control group. All experiments were independently repeated three times.

### 4.10. EdU Cell Proliferation Assay

Cell proliferation was assessed using the EdU-488 Cell Proliferation Detection Kit (RiboBio, Guangzhou, China), with all staining steps carried out protected from light to prevent fluorophore degradation. Sterile 12 mm round coverslips were pre-coated with 0.01% poly-L-lysine at 4 °C overnight, rinsed three times with sterile PBS, and placed into 24-well plates prior to seeding. KGN cells in logarithmic growth phase were harvested by trypsinization, resuspended in complete medium and counted with a hemocytometer. Cells were seeded at a density of 4 × 10^4^ cells per well in 500 μL complete medium, and incubated at 37 °C in a humidified 5% CO_2_ atmosphere for 24 h to allow full adhesion.

After adhesion, the medium was replaced with serum-free DMEM/F12 for 12 h to synchronize cells at the G0/G1 phase. Cells were then exposed to BPS at final concentrations of 0.1 μM, 1 μM, 10 μM, 50 μM and 100 μM, alongside a vehicle control group treated with an equal volume of DMSO. Each group was set up in triplicate wells, and the total treatment time was 24 h. Two hours before the end of incubation, EdU working solution was added to each well to a final concentration of 50 μM, and plates were returned to the incubator for the remaining culture period in the dark.

Following labeling, cells were washed twice with PBS and fixed with 4% paraformaldehyde for 20 min. Residual aldehyde groups were quenched with 2 mg/mL glycine solution for 5 min, after which cells were permeabilized with 0.5% Triton X-100 in PBS for 10 min and washed twice with PBS. The reaction mixture was prepared fresh immediately before use according to the manufacturer’s protocol, added to each well, and incubated for 30 min in the dark, with gentle mixing every 10 min to ensure uniform staining. Cells were then washed three times with PBS for 5 min each, and nuclei were counterstained with 1 μg/mL DAPI for 10 min in the dark, followed by two additional PBS washes.

Coverslips were mounted on glass slides with anti-fade mounting medium and left to dry in the dark for 15 min. Fluorescence images were captured using microscope (Zeiss, Baden-Württemberg, Germany). For each coverslip, five random fields were imaged at 20× magnification with identical exposure settings across all samples. The number of EdU-positive cells and total DAPI-positive nuclei were counted manually by two independent researchers in a blinded fashion, and the cell proliferation rate was expressed as the percentage of EdU-positive cells relative to total cells. All experiments were independently repeated three times.

### 4.11. Western Blotting

Total cellular protein was extracted from KGN cells for immunoblot analysis. RIPA lysis buffer and BCA protein assay kits were purchased from Beyotime Biotechnology (Shanghai, China); SDS-PAGE gel preparation reagents, polyvinylidene difluoride (PVDF) membranes and enhanced chemiluminescence (ECL) substrates were obtained from Millipore (Burlington, MA, USA). Primary antibodies (#4267, #8644, #3027) were sourced from Cell Signaling Technology (Danvers, MA, USA), and horseradish peroxidase (HRP)-conjugated secondary antibodies were provided by Zhongshan Jinqiao Biotechnology (Beijing, China). All protein samples were maintained on ice throughout extraction and processing to minimize proteolytic degradation.

KGN cells in logarithmic growth phase were seeded into 6-well plates at 2 × 10^5^ cells per well in 2 mL complete medium, and cultured at 37 °C in a humidified 5% CO_2_ atmosphere for 24 h until reaching approximately 60% confluence. Cells were then serum-starved for 12 h to synchronize the cell cycle at the G0/G1 phase, followed by exposure to BPS at final concentrations of 0.1 μM, 1 μM, 10 μM, 50 μM and 100 μM. A vehicle control group was set in parallel, receiving an equal volume of DMSO. Cells were harvested at 36 h after BPS administration, with three biological replicates per treatment group.

At a predetermined time point, culture medium was aspirated and cells were rinsed twice with pre-chilled PBS. Cells were lysed on ice for 30 min in 100 μL RIPA buffer freshly supplemented with protease and phosphatase inhibitor cocktails (1:100 dilution), with gentle manual agitation every 10 min to ensure full contact between lysis buffer and adherent cells. Cell lysates were scraped and collected into pre-cooled 1.5 mL centrifuge tubes, then centrifuged at 12,000× *g* for 15 min at 4 °C. The supernatant was carefully transferred to fresh tubes, aliquoted into single-use volumes, and stored at −80 °C to avoid protein damage caused by repeated freeze–thaw cycles.

Protein concentration was determined via the BCA assay following the manufacturer’s protocol. Briefly, bovine serum albumin (BSA) standards and serially diluted samples were loaded into 96-well plates in triplicate, mixed with freshly prepared BCA working solution, and incubated at 37 °C for 30 min protected from light. Absorbance was read at 562 nm using a microplate reader, and sample protein concentrations were calculated against the BSA standard curve.

Equal amounts of total protein (30 μg per lane) were mixed with 5 × SDS loading buffer at a 4:1 volume ratio, denatured by boiling in a water bath for 5 min, and immediately cooled on ice. Proteins were separated by SDS-polyacrylamide gel electrophoresis (SDS-PAGE), with gel concentrations adjusted to the molecular weight of target proteins: 8% gels for proteins larger than 60 kDa, 10% gels for proteins between 30 and 60 kDa, and 12% gels for proteins smaller than 30 kDa. Electrophoresis was run at a constant voltage of 80 V through the 5% stacking gel, then adjusted to 120 V once the bromophenol blue dye front entered the separating gel, and stopped when the dye front reached the bottom edge of the gel.

Proteins were transferred onto PVDF membranes via wet electrotransfer. Gels were equilibrated in pre-chilled transfer buffer for 10 min after electrophoresis. PVDF membranes were activated in anhydrous methanol for 30 s and then equilibrated in transfer buffer for 5 min. The transfer sandwich was assembled from cathode to anode in the order of filter paper, gel, PVDF membrane and filter paper, with air bubbles carefully rolled out between layers to prevent blank areas in protein transfer. Transfer was performed at a constant current of 300 mA for 120 min, with the entire transfer tank placed in an ice bath to avoid overheating and protein degradation. Successful transfer was verified by brief Ponceau S staining, followed by thorough rinsing with deionized water to remove residual stain.

Membranes were blocked in 5% skim milk prepared in Tris-buffered saline containing 0.1% Tween 20 (TBST) for 1.5 h at room temperature on a horizontal shaker. After blocking, membranes were incubated with appropriately diluted primary antibodies overnight at 4 °C with gentle shaking. Used primary antibody solutions were collected and stored at 4 °C for up to three subsequent uses. Membranes were then washed three times with TBST for 5 min each, followed by incubation with corresponding HRP-conjugated secondary antibodies for 1 h at room temperature. Unbound secondary antibodies were removed by three additional 5 min washes with TBST.

Protein bands were visualized using ECL substrate and imaged with a gel documentation system. Band gray values were quantified using ImageJ 1.53g software (National Institutes of Health, Bethesda, MD, USA), with β-actin serving as the endogenous reference protein. The relative expression of each target protein was calculated as the ratio of the target band gray value to that of the corresponding β-actin band from the same sample. All experiments were independently repeated three times.

### 4.12. Statistical Analysis

All statistical analyses and data visualization were carried out using GraphPad Prism 9. Quantitative results are expressed as mean ± standard deviation (mean ± SD). All in vitro cellular experiments were independently repeated three times as biological replicates. For assays containing technical replicates (e.g., replicate wells in CCK-8 assays, duplicate sample loading for protein quantification), intra-assay means were calculated per biological replicate first, and final statistical tests were performed on the means of independent biological replicates to eliminate false positives caused by technical replication.

For multi-group comparisons covering the vehicle control and five BPS concentration groups at a single treatment time point, one-way analysis of variance (one-way ANOVA) was first conducted to evaluate the overall difference across all groups. When the overall group effect was statistically significant (*p* < 0.05), Tukey’s post hoc test was applied for subsequent pairwise multiple comparisons. For comparisons between two independent sample sets, a two-tailed independent samples *t*-test was adopted. *, *p* < 0.05; **, *p* < 0.01; ***, *p* < 0.001; ****, *p* < 0.0001.

## 5. Conclusions

By integrating network toxicology, molecular docking, all-atom molecular dynamics simulations, and in vitro cellular assays, this study systematically delineated the potential molecular mechanisms linking BPS exposure to PCOS pathogenesis. We identified 199 overlapping target genes common to both BPS biological action and PCOS disease progression, Functional enrichment analyses of the top 30 predicted core hub genes indicated that BPS-related actions are predominantly involved in oxidative stress, hormonal response, insulin response, inflammatory regulation and cell-cycle control, with significant enrichment in the PI3K-Akt, FoxO, AGE-RAGE and endocrine resistance signaling pathways. Molecular docking simulations demonstrated that most of the top 15 predicted core hub proteins exhibited Vina-calculated binding free energies below −5.0 kcal/mol, a threshold widely accepted for stable ligand–receptor complex formation. Given their prominent topological centrality in the PPI network, as well as favorable binding affinities for BPS and stable binding poses validated by molecular docking assays, EGFR, ESR1, and IGF1 were prioritized as core candidate hub targets. The 100 ns all-atom molecular dynamics simulations demonstrate that BPS binding induces only minor conformational perturbations in EGFR and ESR1, with negligible alterations to backbone stability, core domain flexibility, global compactness and surface properties, whereas it effectively dampens global backbone fluctuations and residue-level mobility of IGF1, with intermolecular hydrogen bond strength following the order IGF1 > EGFR > ESR1. Subsequent cellular validation further supports EGFR, ESR1, and IGF1 as functionally relevant targets through which BPS exerts its biological effects. Collectively, these findings advance our mechanistic understanding of BPS-mediated reproductive toxicity, and provide a structural and biological framework for the safety evaluation of bisphenol alternatives and future research into environmental risk factors for PCOS.

## Figures and Tables

**Table 1:** Detailed information about BPS molecule (National Center for Biotechnology Information. PubChem Compound Summary for CID 6626, Bisphenol S; accessed on 15 October 2025, from https://pubchem.ncbi.nlm.nih.gov/compound/6626).

Property Name	Property Value	Reference
Molecular weight	250.27 g/mol	Computed by PubChem 2.2 (PubChem release 15 September 2025)
Molecular formula	C_12_H_10_O_4_S	Computed by PubChem 2.2 (PubChem release 15 September 2025)
SMILES	C1=CC(=CC=C1O)S(=O)(=O) C2=CC=C(C=C2)O	Computed by OEChem 4.2.0 (PubChem release 15 September 2025)

## Data Availability

The original contributions presented in this study are included in the article/Supplementary Materials. Further inquiries can be directed to the corresponding authors.

## Supplementary Materials

The following supporting information can be downloaded at: https://www.mdpi.com/article/10.3390/ijms27208961/s1.

## Abbreviations

The following abbreviations are used in this manuscript:

BPS	Bisphenol S
PCOS	Polycystic ovary syndrome
HPO	hypothalamic–pituitary–ovarian
EDCs	Endocrine-disrupting chemicals
BPA	Bisphenol A
PPI	Protein–protein interaction
GO	Gene ontology
KEGG	Kyoto Encyclopedia of Genes and Genomes
HPA	Human Protein Atlas
BP	Biological process
CC	Cellular component
MF	Molecular function
MD	Molecular dynamics
RMSD	Root-mean-square deviation
