# Supplementary material for: Integrated Network Toxicology, Molecular Dynamics Simulation and In Vitro Assays Identify EGFR, ESR1 and IGF1 as Core Mediators of Bisphenol S-Exacerbated Polycystic Ovary Syndrome
Source: Int J Mol Sci. 2026 Oct 9;27(20):8961. doi: 10.3390/ijms27208961 (PMC13704305; doi:10.3390/ijms27208961)
Supplement: Supplementary file 1 [file ijms-27-08961-s001.zip › ijms-4553887-supplementary.pdf]

ERS1

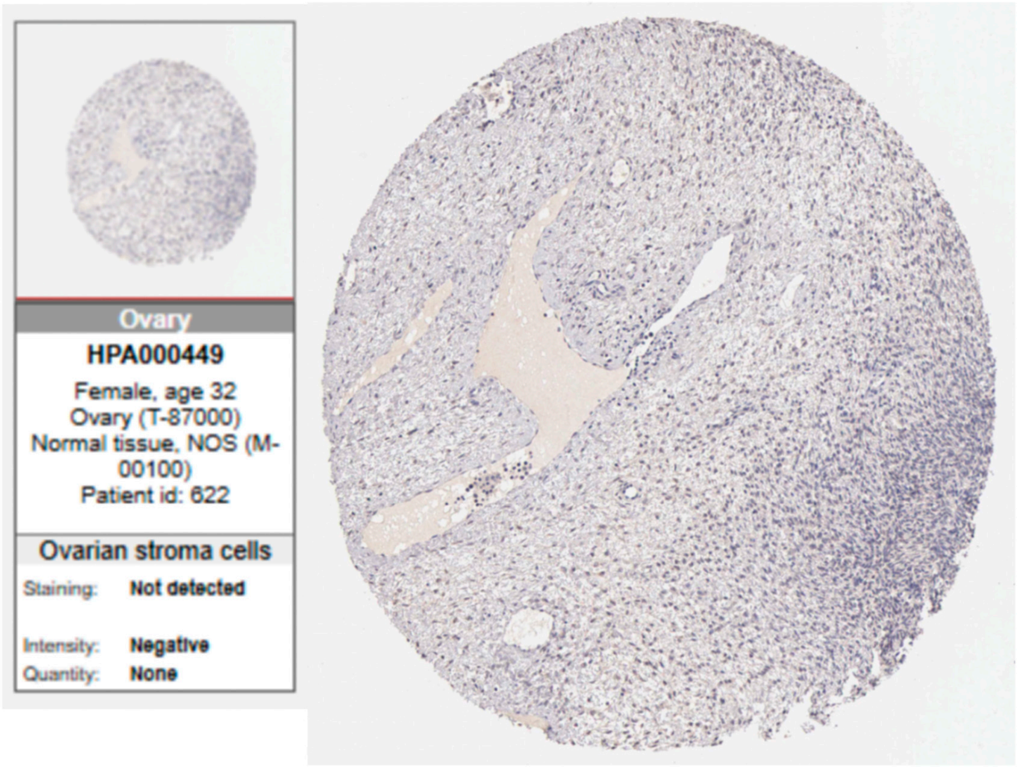

Figure S1. Immunohistochemical (IHC) expression profiles of ERS1 protein in normal ovarian tissues (data from the HPA database)

EGFR

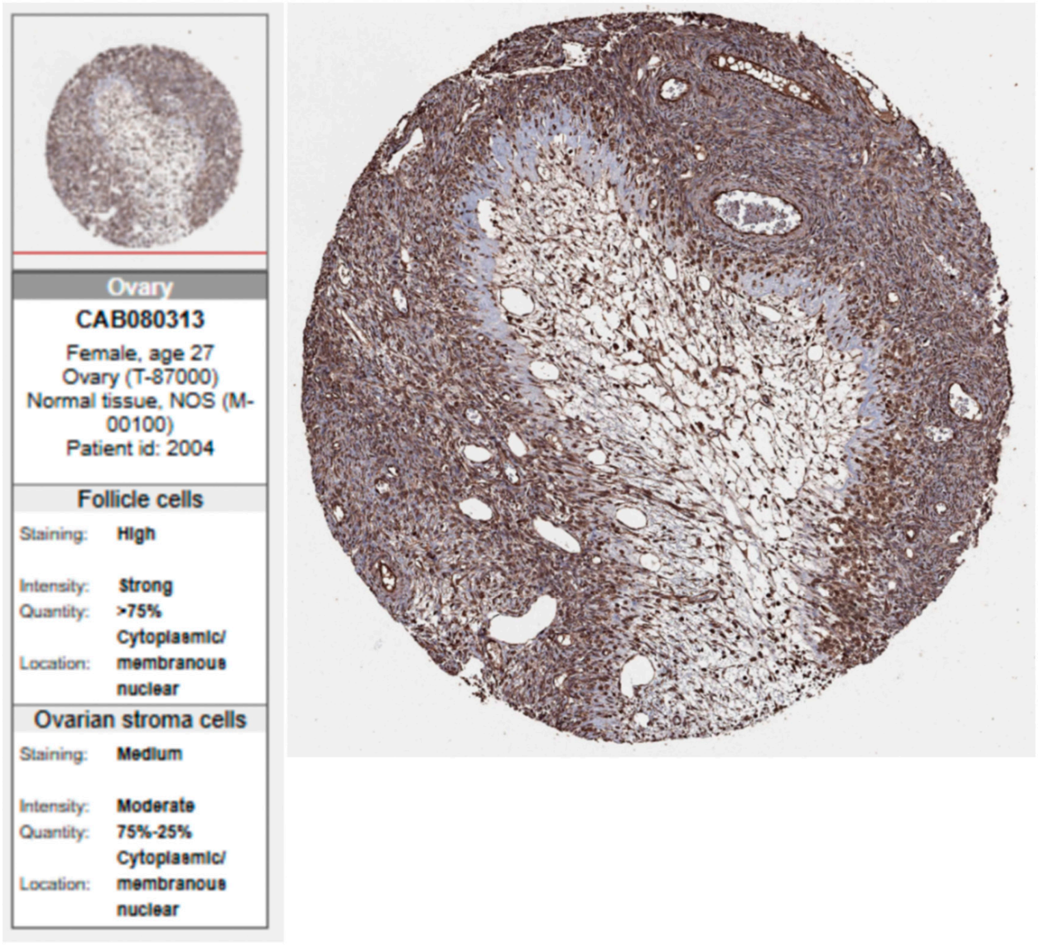

Figure S2. Immunohistochemical (IHC) expression profiles of EGFR protein in normal ovarian tissues (data from the HPA database)

IGF1

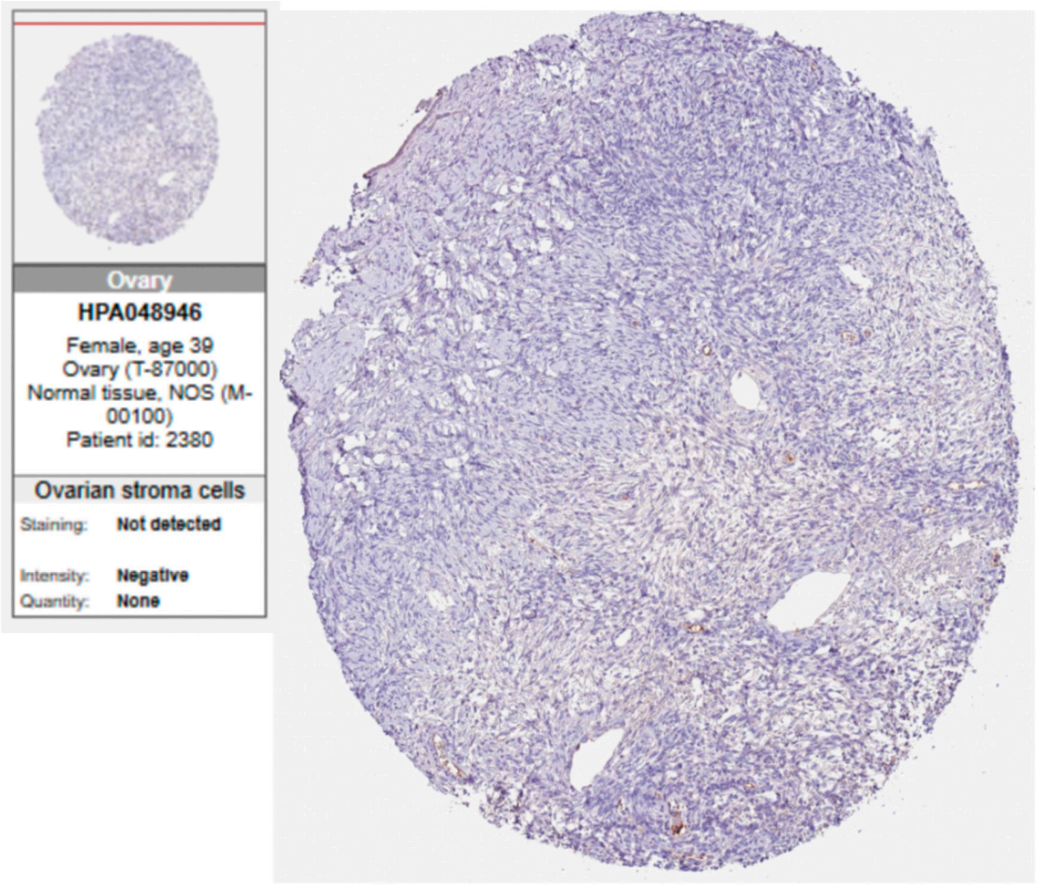

Figure S3. Immunohistochemical (IHC) expression profiles of IGF1 protein in normal ovarian tissues (data from the HPA database)
